# Environmental Predictors of US County Mortality Patterns on a National Basis

**DOI:** 10.1371/journal.pone.0137832

**Published:** 2015-12-02

**Authors:** Melissa P. L. Chan, Robert S. Weinhold, Reuben Thomas, Julia M. Gohlke, Christopher J. Portier

**Affiliations:** 1 Environmental Sciences Program, Southern Illinois University Edwardsville, Edwardsville, IL, 62026, United States of America; 2 Independent Researcher and Journalist, Colorado City, CO, 81019, United States of America; 3 School of Public Health, University of California, Berkeley, CA, 85736, United States of America; 4 School of Public Health, University of Alabama, Birmingham, AL, 35294, United States of America; 5 National Center for Environmental Health and Agency for Toxic Substances and Disease Registry, U.S. Centers for Disease and Prevention, Atlanta, GA 30341, United States of America; University of Jyväskylä, FINLAND

## Abstract

A growing body of evidence has found that mortality rates are positively correlated with social inequalities, air pollution, elevated ambient temperature, availability of medical care and other factors. This study develops a model to predict the mortality rates for different diseases by county across the US. The model is applied to predict changes in mortality caused by changing environmental factors. A total of 3,110 counties in the US, excluding Alaska and Hawaii, were studied. A subset of 519 counties from the 3,110 counties was chosen by using systematic random sampling and these samples were used to validate the model. Step-wise and linear regression analyses were used to estimate the ability of environmental pollutants, socio-economic factors and other factors to explain variations in county-specific mortality rates for cardiovascular diseases, cancers, chronic obstructive pulmonary disease (COPD), all causes combined and lifespan across five population density groups. The estimated models fit adequately for all mortality outcomes for all population density groups and, adequately predicted risks for the 519 validation counties. This study suggests that, at local county levels, average ozone (0.07 ppm) is the most important environmental predictor of mortality. The analysis also illustrates the complex inter-relationships of multiple factors that influence mortality and lifespan, and suggests the need for a better understanding of the pathways through which these factors, mortality, and lifespan are related at the community level.

## Introduction

There is a growing body of evidence that outdoor air pollution and socioeconomic status are associated with cardiorespiratory and cardiovascular diseases [1–5] and the combined effects of disparities in health-related behaviors, environmental conditions, social structures, and the contact and delivery of health care. A relationship between income inequality, social capital, primary care and health outcomes has been examined in several studies, but few published analyses included all variables simultaneously [6–8].

Over the past decades, socioeconomic health disparities have widened in the general populations of the US and Europe [7, 9–11] though great attention is being given to racial and ethnic disparities in health care [12]. Race and class are both independently associated with health status, although it is often difficult to disentangle the individual effects of the two factors [12]. In the US, income, certain races and some ethnic groups are found to be associated with poorer population health [12–15]. Past analyses indicated that income inequality and morbidity or mortality have been complicated by existing racial/ethnic and age differences in income and mortality prospects [16]. Income inequality shows a more powerful effect on health when a race variable is added, and when both race and urbanization terms are entered [17–20]. Differences in mortality and morbidity rates are partly attributable to the fact that people in the upper socioeconomic levels have healthier behaviors and lifestyles compared to people in the lower levels, in which people in the latter group die earlier than do people at the former group, a pattern that holds true in a progressive fashion from the poorest to the richest [21–23]. Though community social capital is not related to all-cause mortality; lower mortality risks for cancer and suicide are found in socially strong neighborhoods compared with socially weak neighborhoods [24]. Some researchers suggest that education is the critical variable since better-educated people are more likely to acquire better jobs and to achieve higher social status [24–26]. Several studies have found no association between community social capital and health [27] whereas others have reported positive association [28–36]. Social capital has been linked to connections among individuals-social networks and the norms of reciprocity and trustworthiness that arise from them [37]. Social capital is defined as norms and networks that facilitate collective action [38–39].

Numerous epidemiological studies have shown association of acute and chronic exposures to airborne particles with risk for adverse effects on morbidity and mortality [40–46]. Short-term exposure to PM_2.5_ increases the risk for hospital admission for cardiovascular and respiratory diseases. Cardiovascular risks tended to be higher in counties located in the Eastern region of the US, which included the Northeast, the Southeast, the Midwest, and the South [47–50].

This study develops a model to predict the mortality rates for different diseases by county across the US taking into account various factors such as environmental pollutants, weather, socioeconomic factors, social capital and other factors. The developed model is then applied to predict changes in mortality caused by changing environmental pollutant factors.

## Materials and Methods

### 2.1. Definitions of the groupings

Only 3,110 of the total 3,141 counties within the continental US excluding Alaska and Hawaii were used in our study; 31 were excluded due to lack of reliable death rates, low-population density or lack of environmental, socioeconomic, social capital or other data. Analyses of the data were conducted using grouping either by region or by population density. Five population density groups are presented here. Grouping is defined as the group sorted according to the population density with the first grouping consisting of counties with the smallest population density and the last grouping consisting of counties with the largest population density. Several different density groupings were considered. The analysis for all counties combined with no groupings showed considerable lack of fit and is not discussed further. The analysis grouping counties by region of the country was considerably better than the full analysis, but sufficiently worse than the groupings by population density and also will not be discussed further.

### 2.2. Data Sources for Population, Mortality Rates and Health Outcomes

Table 1 presents the summary of the data sources, data year(s) and the corresponding references used in the present study. The variables used in this study have been carefully selected and identified in the literature as likely to affect the mortality rates of different diseases. County-specific mortality data were obtained from the Centers for Disease Control and Prevention (CDC) WONDER/PC Software (http://wonder.cdc.gov/). The data were standardized for age using the 1999–2002 US population as the reference population. Mortality has been known as one of the most commonly used health status indicators, especially in studies on income equality and health. The primary cause of death was classified according to the International Classification of Diseases, Tenth Revision (ICD-10) codes. The following causes of death were distinguished: (i) all causes mortality, (ii) cardiovascular diseases, (iii) cancers, (iv) chronic obstructive pulmonary disease (COPD), and (v) the combination of (ii)-(iv). Life expectancy was also evaluated. Cardiovascular diseases diagnosed as primary cause of death were acute rheumatic fever (ICD I00-I02), chronic rheumatic heart diseases and hypertensive diseases (ICD I05-I15), ischemic heart diseases and pulmonary heart and circulation diseases (ICD I20-I28), other forms of heart disease (ICD I30-I52), diseases of arteries, arterioles, capillaries, veins, lymphatic vessels, and lymph nodes not covered elsewhere (ICD I70-I89), other or unspecified disorders of the circulatory system (ICD I95-I99) and stroke (ICD I60-69). Cancers included colon (ICD C18), pancreas (ICD C25), trachea, bronchus and lung (ICD C33-C34), breast (ICD C50), ovary (ICD C56), prostate (ICD C61), bladder (ICD C67), brain, spinal cord, cranial nerves and other central nervous system (ICD C71-C72), non-Hodgkin’s lymphoma (ICD C82-C85) and multiple myeloma including leukemia (ICD C90-C95). COPD included chronic lower respiratory diseases, bronchitis and emphysema (ICD J40-J44 and ICD J47). Sex-adjusted life expectancy data were obtained from the US Department of Health and Human Services, Office on Women’s Health [51].

**Table 1:** Data Sources for Each County.

Data	Data Source	Data Year(s)	Reference Number
County-specific mortality rate	CDC WONDER	1999–2002	—
Life expectancy	US Department of Health and Human Services	1999	51
Weather	National Climatic Data Center (NCDC)	2002	52
Environmental pollutants (NO_x_, SO_x_, PM_2.5_, PM_10_, VOC, NH_3_, CO)	US EPA National Emission Inventory	2002	53
Environmental pollutant (Ozone)	US EPA Air Quality Monitoring Information	2007	54
Environmental pollutant (Diesel)	US EPA National Air Toxics Assessment	1996	55
Social capital	Rupasingha and Goetz	1997	56
Risk factors	US DHHS Community Health Status Indicators	2009	57
Socio-economic	US Census Bureau	2000	58

Monthly averages of three primary determinants of weather: temperature, precipitation, total heating degree days and total cooling degree days were obtained from the National Climatic Data Center (NCDC) [52]. Data for nitrogen oxides (NO_x_), sulfur oxides (SO_x_), particulate matter with aerodynamic diameter < 2.5 μm (PM_2.5_), particulate matter with aerodynamic diameter < 10 μm (PM_10_), volatile organic compounds (VOCs), ammonia (NH_3_) and carbon monoxide (CO), were obtained from the US Environmental Protection Agency’s (EPA) National Emission Inventory [53]. Ozone data were obtained from US EPA’s Air Quality Monitoring Information [54]. The emission data from diesel were obtained from the US EPA’s National Air Toxics Assessment [55].

County-level social capital data were retrieved from Rupasingha and Goetz [56] and also described elsewhere. In brief, his social capital measures are based upon 14 county-level indicators derived from various sources to assess different facets of social capital which are divided into five core components such as community organizational life, engagement in public affairs, community voluntarism, informal sociability and social trust. In our study, sixteen individual-level social capital indicators were analyzed, corresponding to numbers of (1) bowling centers, (2) civic and social organizations, (3) physical fitness facilities, (4) public golf courses, (5) religious organizations, (6) sports clubs, managers and promoter, (7) memberships in sports and recreation clubs, (8) political organizations, (9) professional organizations, (10) business organizations, (11) labor organizations, (12) memberships in organizations not elsewhere classified, (12) votes cast for President in 1996, and (13) non-profit organizations. We also included a variable aggregating (1)-(12) and a variable linked to the response rate (mail in) from the 2000 Census.

Risk factors such as percentages of population with (1) no exercise, (2) few fruits and vegetables, (3) obesity, (4) high blood pressure, (5) smoking) and (6) diabetes were obtained from the US Department of Health and Human Services’ Community Health Status Indicators [57].

County-level descriptor variables were divided into two categories, determinants related to health and determinants related to wealth. These data were retrieved from the US Census Bureau [58]. Potential health determinants included were poverty, education level, number of primary care physicians per 10,000 population, number of dentists per 10,000 population, racial composition, median ages for each sex and combination of both sexes. The wealth of the counties was characterized by median household income, median family income and county per capita income. Crime characteristics, housing characteristics and employment by industry (agriculture, fishing, mining, construction and other outdoor related jobs) for both sexes per 10,000 population were used as an indicator to evaluate any association between these variables and mortality.

We used different sources of data because not all data were available for the same year. The period of the data that we chose was as close to each other’s period as possible to minimize large difference. The difference of the data from one year to another year for any particular variable used in the model, if available, was less than 1%. Also, most of the variables that we used in the study are slow moving in the time dimension. Hence, it was concluded that the period of the data being used and any bias induced by the heterogeneity in sampling time, which is believed to be small, did not have any major impact in our analyses.

More than 99% of the data were available for all counties for the variables that we used in the study except for ozone. According to the US EPA, ozone is not measured in counties known to be in compliance (that is, with low values). For counties with missing data for a specific variable, data from the three closest counties regardless of population density were averaged to impute a value under the assumption that these counties demonstrate similar or almost similar characteristics with the three closest counties.

### 2.3 Statistical Analysis

The 3,110 counties were sorted according to population density in ascending order. These counties were divided into two sets by using systematic random sampling to ensure an equal distribution of population sizes in both samples by assigning every sixth county in Set 2 after choosing a random starting point in the sorted set of counties. Set 1, consisting of 2,591 counties, was used for estimation of model parameters for predicting the mortality rates of each disease. Set 2, consisting of 519 counties was used to validate the resulting model.

The regression model being used in the analysis assumes that:

$$E\left(Y_{ij}\right)=\mu +\overset{M}{\underset{m=1}{\sum }}\beta_{m}X_{mi}+\overset{K}{\underset{k=1}{\sum }}\alpha_{kj}C_{ki}$$

where:

*μ* = the average of variable *Y* over all counties

*β*_*m*_ = the slope of the response as a function of environmental variable *m* (*β*_*m*_≤0 for life expectancy and *β*_*m*_≥0 for all other environmental variables)

*X*_*mi*_ = the value of environmental variable *m* for county *i*

*α*_*kj*_ = the slope of the response as a function of non-environmental variable *k* for counties in county group *j*

*C*_*ki*_ = the value of non-environmental variable *k* for county *i*

A modified stepwise regression was used for the analysis with all of the environmental variables included in the original analysis without non-environmental variables. The algorithm was designed to maximize the inclusion of environmental variables in the final model. Hence, we chose to use a smaller p-value (p = 0.03) in our analysis. (1) The slopes of all environmental factors were evaluated for significance and the least significant (p>0.03) was removed from the analysis (backwards algorithm). This step was repeated until only significant environmental variables remained. (2) The non-environmental variables were each added into the regression to determine which was most significant; this variable was added to the model (forward algorithm). This was repeated until there were no new significant non-environmental variables to be included in the model (p<0.03). (3) The forward algorithm was then applied to the environmental variables. If new variables entered the regression, a backwards algorithm was applied to the non-environmental variables to remove any that were no longer significant. If no changes occurred, the algorithm stopped. If changes occurred, the process was repeated starting with (2). Since we were interested in the harmful effects of environmental pollutants, their slope was restricted to be less than 0 for life expectancy and greater than zero for mortality. Only those variables that were significantly different from zero and in the direction of harm were included. Analyses were done without using this restriction, but the resulting improvements in health from increased air pollution could not be supported by other studies and the restriction was added to avoid false interpretations. In terms of the quality of the overall fit, this restriction had no overall impact.

Regression parameters estimated from Set 1 were used to predict the mortality rates of each disease and life expectancy for counties in each region in Set 2. Residual plots were used to determine how well a particular model fit the data, identifying outlying observations and suggesting terms missing from the linear predictor. All data were normalized prior to analysis.

When the optimal models were obtained, they were used to predict the impact of the environmental variables on mortality for each county. All environmental pollutants were reduced to the national 25^th^ percentile under the assumption that counties should be able to achieve this number. In another scenario, considering that counties with high density populations, particularly urban counties, may not be able to achieve the national 25^th^ percentile, all environmental pollutants were reduced to the group 25^th^ percentile within each group defined by population density. All analyses were conducted in Matlab (Version 7.7.0.471).

## Results

### 3.1. Demographic characteristics

Demographic characteristics of the 3,110 counties from five population density groups are outlined in S1–S7 Tables. The availability of primary care ranged from 0 per 100,000 population to 581.2 per 100,000 population. There was no distinct geographical pattern in the availability of primary care. The percentage of males with at least a bachelor degree ranged from 0% to 70.6%, whereas the percentage of females with at least a bachelor degree ranged from 3.9% to 57.7%. The percentage of the population in poverty ranged from 0% to 56.9%. Generally, lower poverty counties were located in the northern region of the country whereas higher poverty counties were located in the southern region of the country. The western region has lower poverty than the eastern region.

### 3.2. Mortality

S8 Table shows the average mortality rates per 100,000 population per year for the different diseases in five population density groups. The total mortality rate from all-causes across all counties in the US ranged from 375.2 to 1,799.2. The mortality rate for cardiovascular diseases ranged from 113.4 to 640.6. The lowest rates for cardiovascular diseases were found in the Northeast, upper Midwest and the western half of the country excluding Nevada and some of inland California whereas the highest rates were located in Appalachia, the Southeast and states bordering Lake Erie. The mortality rate for cancers ranged from 0.0 to 314.9. The lowest cancer mortality rates were in the Rocky Mountain region. Almost the entire eastern half of the country had high cancer mortality rates with the worst in the Southeast. The Northeast, the southern two-thirds of Florida and the southern half of the Rocky Mountain had the lowest cancer mortality rates whereas the highest cancer mortality rates were found primarily in most of the Southeast regions especially the belt including Arkansas, Tennessee, Virginia and the Carolinas. The mortality rate for COPD ranged from 0.0 to 135.4. The lowest COPD rates were located in the upper Midwest, Utah and coastally-influenced zones of the Northeast and mid-Atlantic region whereas the highest COPD rates were dominant in the Rocky Mountain region, west Texas, Nevada, inland California and inland areas of the lower Midwest, Appalachia and Southeast. Life expectancy ranged from 66.6 years to 81.3 years. Generally, counties in South Dakota have the lowest life expectancy and counties in Colorado have the highest life expectancy.

### 3.3. Statistical Analysis

To conserve space, Table 2 presents the regression parameters that entered four or five quintiles for life expectancy, all-causes mortality and cardiovascular diseases for the case where the counties are grouped into five groups consisting of: (1) the 1/5 of counties with the lowest population densities; (2) the 1/5 of the counties with mid-range population densities described as Quintiles 2, 3 and 4; and (3) the 1/5 of the counties with the highest population densities. S9–S14 Tables present the details of the regression parameters for the life expectancy; all-causes mortality; cardiovascular diseases; combination of cardiovascular diseases, cancers and COPD; cancers and COPD.

**Table 2:** Regression Parameters Derived from Stepwise Regression Analysis of Variables for Life Expectancy, All-Causes Mortality and Cardiovascular Diseases for Five Population Density Groups.

Variable		Lowest Density Quintile			Quintile 2			Quintile 3			Quintile 4			Highest Density Quintile		
		Regression coefficient	Standard deviation	P value	Regression coefficient	Standard deviation	P value	Regression coefficient	Standard deviation	P value	Regression coefficient	Standard deviation	P value	Regression coefficient	Standard deviation	P value
Intercept Term	Life expectancy							78.58	0.322	0						
	All-causes							685.1	19.95	0						
	Cardiovascular diseases							318.6	11.04	0						
Ozone	Life expectancy							-0.1288	0.02404	9.20E-08						
	All-causes							13.32	1.721	1.44E-14						
	Cardiovascular diseases							7.552	0.9862	2.67E-14						
PM_10_	Life expectancy															
	All-causes															
	Cardiovascular diseases							1.726	0.7639	0.02394						
% Single parent households	Life expectancy	-0.366	0.05238	3.55E-12	-0.2809	0.09693	0.003785	-0.6453	0.06914	0	-0.6837	0.07966	0			
	All causes	53.09	3.392	0	57.45	4.91	0	25.19	6	2.78E-05	38.74	4.997	1.31E-14	26.9	5.525	1.20E-06
	Cardiovascular diseases	16.6	3.503	2.26E-06	10.74	2.778	0.0001135	15.74	2.72	8.18E-09	9.347	3.116	0.002732	8.379	3.068	0.006357
% 16–64 years (Both sexes) with physical disability	Life expectancy				-0.2238	0.04946	6.31E-06	-0.154	0.05502	0.005179	-0.2219	0.06478	0.0006252	-0.8324	0.08994	0
	All causes	12.92	3.005	1.78E-05	17.36	3.628	1.80E-06	12.98	3.896	0.0008784	18.52	4.623	6.34E-05	52.76	6.951	4.46E-14
	Cardiovascular diseases															
% Votes cast for President	Life expectancy							0.2218	0.06212	0.0003633	0.4051	0.0561	6.82E-13	0.3019	0.06429	2.80E-06
	All causes	-21.49	3.209	2.64E-11	-21.52	4.234	3.99E-07				-28.54	3.404	1.11E-16	-19.14	3.692	2.34E-07
	Cardiovascular diseases	-11.26	1.953	9.12E-09	-12.15	2.294	1.27E-07				-9.241	2.301	6.07E-05	-7.698	2.21	0.0005037
% Male with at least a bachelor degree	Life expectancy	0.2307	0.06712	0.0005969				0.6022	0.09428	2.01E-10						
	All causes							-57.53	5.082	0						
	Cardiovascular diseases	-18.59	2.921	2.33E-10	-13.08	3.137	3.16E-05	-15.84	3.986	7.27E-05				-14.52	1.754	2.22E-16
% Hispanic or Latino	Life expectancy				0.4485	0.06335	1.87E-12									
	All causes	-18.36	2.409	3.59E-14	-32.48	4.157	8.11E-15	-48.55	4.605	0	-20.81	7.194	0.003854			
	Cardiovascular diseases	-7.496	1.803	3.34E-05	-11.89	3.154	0.0001683	-17.68	2.477	1.25E-12	-24.47	3.147	1.08E-14	-21.42	3.034	2.12E-12
% Adults reporting no exercise	Life expectancy				-0.3332	0.0512	9.14E-11	-0.2434	0.04958	9.75E-07	-0.1769	0.05927	0.002861	-0.2984	0.05916	4.88E-07
	All causes	10.67	3.972	0.007298	11.19	3.974	0.004909	12.62	3.639	0.0005326	16.35	3.974	4.00E-05			
	Cardiovascular diseases	13.81	2.306	2.41E-09	9.639	2.173	9.58E-06	6.876	1.986	0.000543	8.057	2.201	0.000257	8.173	2.618	0.001821
% Adults reporting high blood pressure	Life expectancy	-0.2747	0.06334	1.50E-05	-0.1363	0.04689	0.003684	-0.1435	0.04974	0.003939	-0.136	0.04949	0.006039			
	All causes										9.657	3.519	0.006112			
	Cardiovascular diseases				5.142	1.88	0.006277							-5.812	2.203	0.008381
Total suicide death per 100,000 population	Life expectancy							-0.1464	0.05673	0.009927	-0.2905	0.07842	0.000217			
	All causes	12.48	1.863	2.57E-11	13.13	3.516	0.0001921	13.7	4.074	0.0007833	21.39	5.681	0.0001702			
	Cardiovascular diseases	5.224	1.071	1.13E-06												

**Fig 1:**
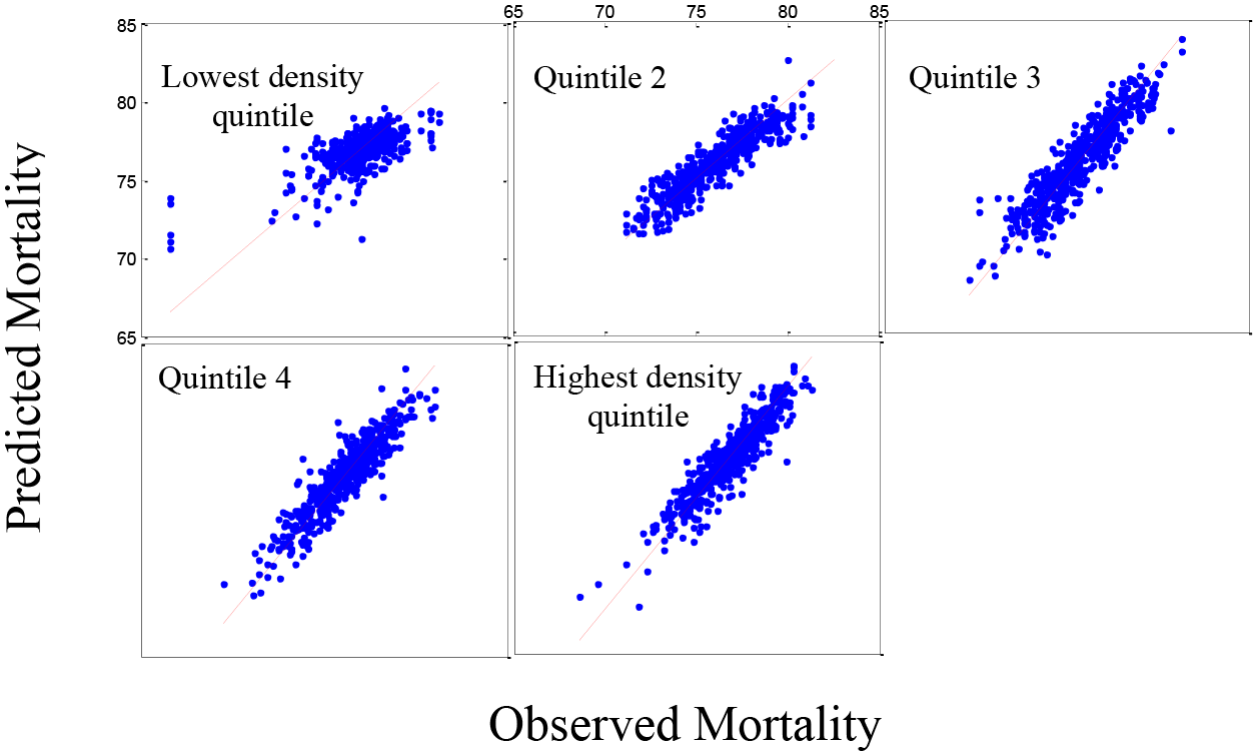
Set 1: The estimated mortality plot for life expectancy. Observed versus estimated mortality in 2,591 counties in the prediction set (Set 1) using stepwise regression for five population density groups (R-squared = 0.7921).

**Fig 2:**
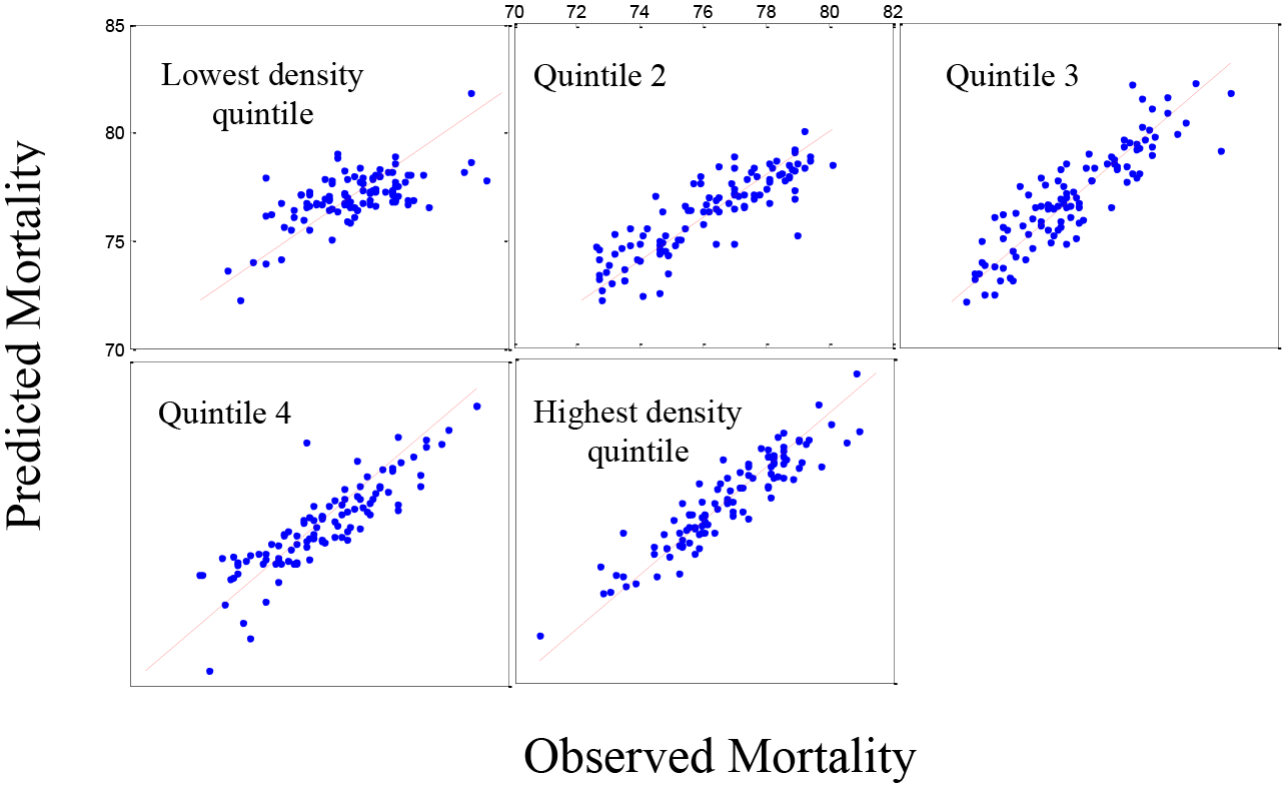
Set 2: The estimated mortality plot for life expectancy. Observed versus estimated mortality in 519 counties in the validation set (Set 2) using stepwise regression for five population density groups.

The analysis of life expectancy for five population density groups (Table 2) showed only four social/economic predictors were significant in four groups (percentage of single parent households, percentage of people aged 16–64 years with physical disability, percentage of adults reporting no exercise and percentage of adults reporting high blood pressure). Of the environmental variables, life expectancy decreased with ozone (p<0.001). Fig 1 shows the predicted model versus the observed data for life expectancy in the five population density groups. Based on the residual plots (not shown), there is a slight lack-of-fit in the model, under-predicting the higher mortality rates and over predicting the lower mortality rates. However, the R-squared values for the plots are acceptable (0.7921). Fig 2 shows the same pattern for the 519 counties in the validation set and a similar, although less striking, lack-of-fit is observed.

For all-causes mortality, only two social/economic predictors (percentage of single parent households, percentage of people aged 16–64 years with physical disability) were significant in all groups and four were significant (p<0.001) in four population density groups (percentage of votes cast for President, percentage of Hispanic or Latino, percentage of adults reporting no exercise, total suicide death per 100,000 population). Of all of the environmental variables, only ozone was significant (p<0.001).

**Fig 3:**
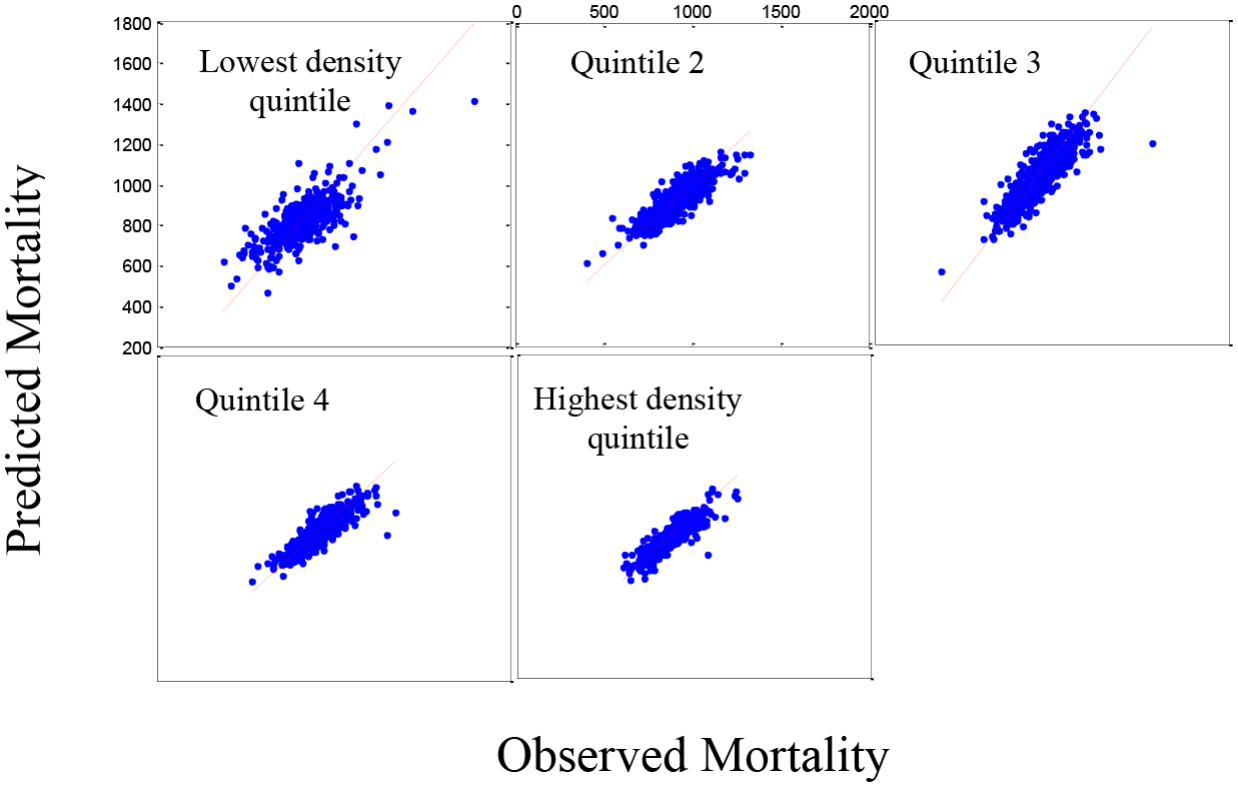
Set 1: The estimated mortality plot for all causes mortality. Observed versus estimated mortality in 2,591 counties in the prediction set (Set 1) using stepwise regression for five density population groups (R-squared = 0.7417).

**Fig 4:**
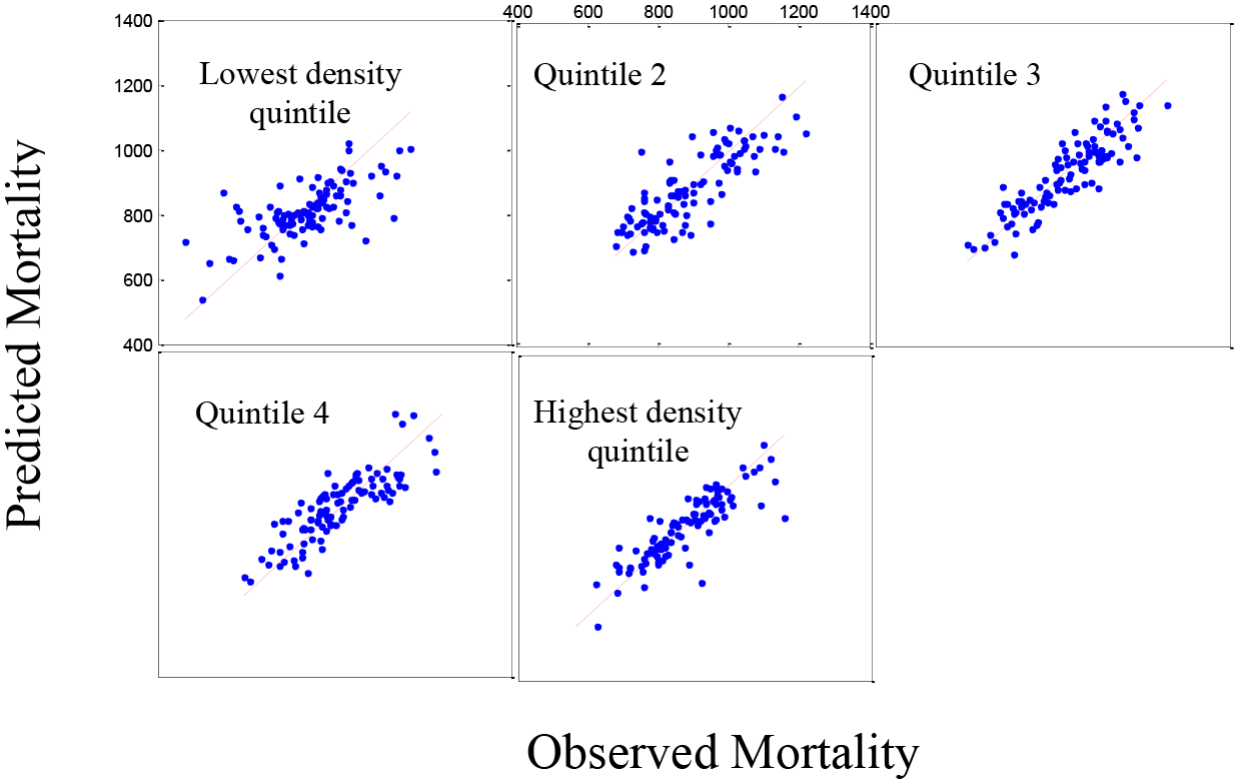
Set 2: The estimated mortality plot for all causes mortality. Observed versus estimated mortality in 519 counties in the validation set (Set 2) using stepwise regression for five density population groups.

Fig 3 shows the fit for all-causes mortality in the five population density groups where there is still a slight lack-of-fit in the model, again under-predicting the higher mortality rates and over predicting the lower mortality rates based on the residual plots (not shown). However, the R-squared values for the plots are clearly acceptable (0.7417). Fig 4 shows the same pattern for the 519 counties in the validation set.

For cardiovascular diseases, one social/economic predictor decreased mortality significantly (percentage of Hispanic or Latino) and two increased mortality significantly (percentage of single parent households, percentage of adults reporting no exercise) (p<0.001) in all groups. Two were significant (p<0.001) in four population density groups with two significant in the highest population density groups (percentage of votes cast for President, percentage of males with at least a bachelor degree). Ozone (p<0.001) and PM_10_ (p = 0.02) entered into the regression model and significantly increased mortality in all population density groups.

**Fig 5:**
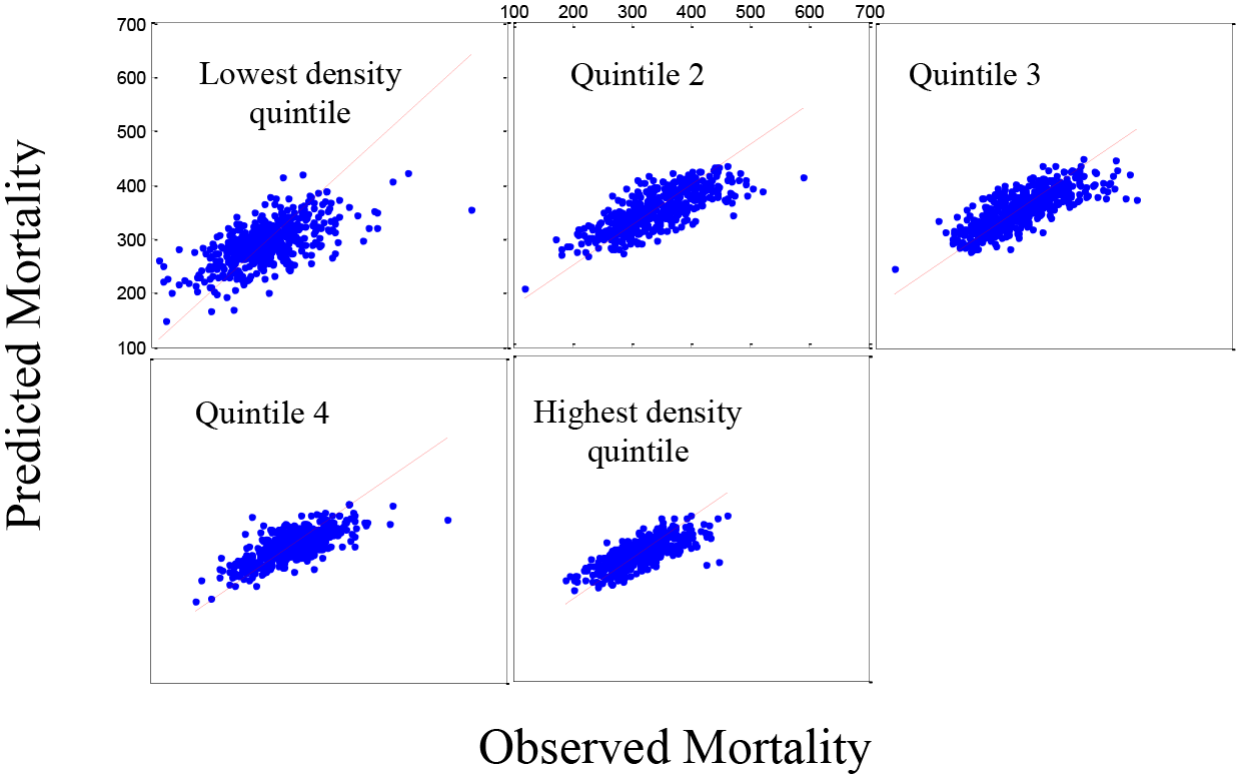
Set 1: The estimated mortality plot for cardiovascular diseases. Observed versus estimated mortality in 2,591 counties in the prediction set (Set 1) using stepwise regression for five density population groups (R-squared = 0.5883).

**Fig 6:**
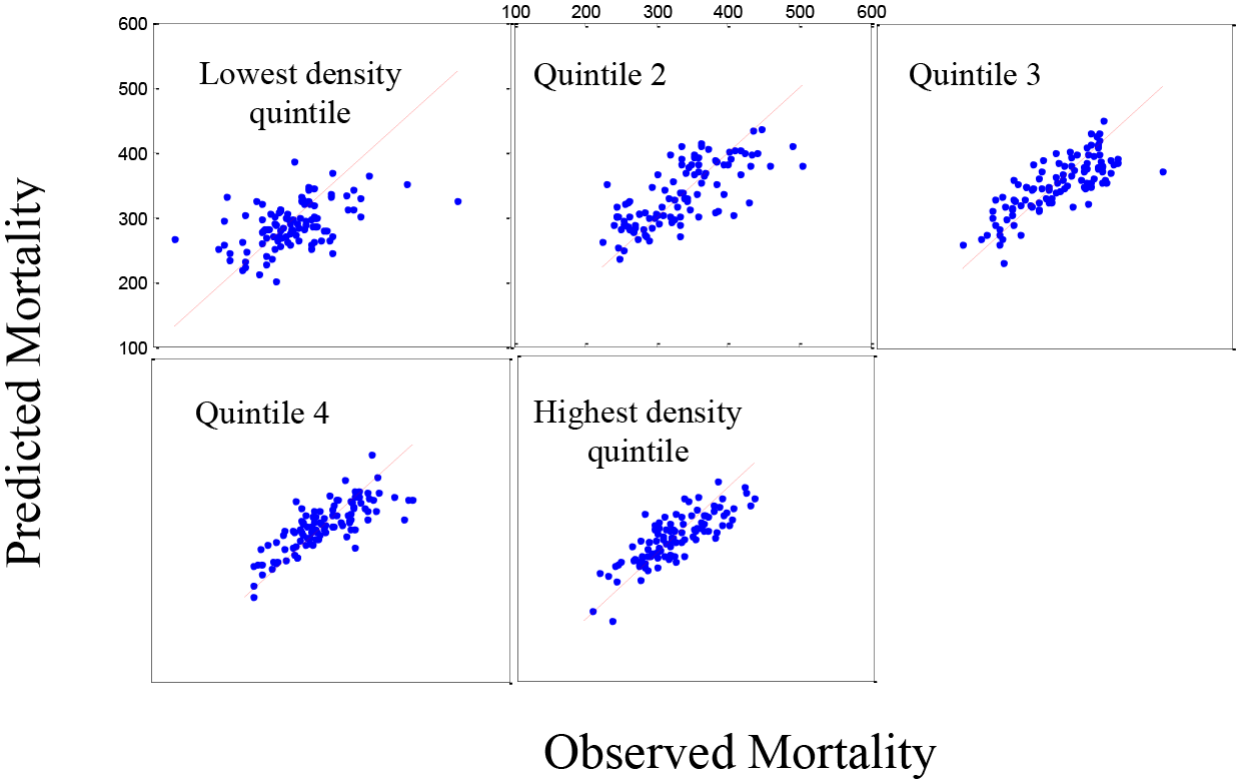
Set 2: The estimated mortality plot for cardiovascular diseases. Observed versus estimated mortality in 519 counties in the validation set (Set 2) using stepwise regression for five density population groups.

Fig 5 shows the predicted model versus the observed data for cardiovascular diseases in the five population density groups. Based on the residual plots (not shown), there is a slight lack-of-fit in the model, under-predicting the higher mortality rates and over predicting the lower mortality rates (R-squared = 0.5883). Fig 6 shows the same pattern for the 519 counties in the validation set. S1–S6 Figs present the predicted model versus the observed data for four major causes of mortality, cancers and COPD respectively for five population density groups.

**Fig 7:**
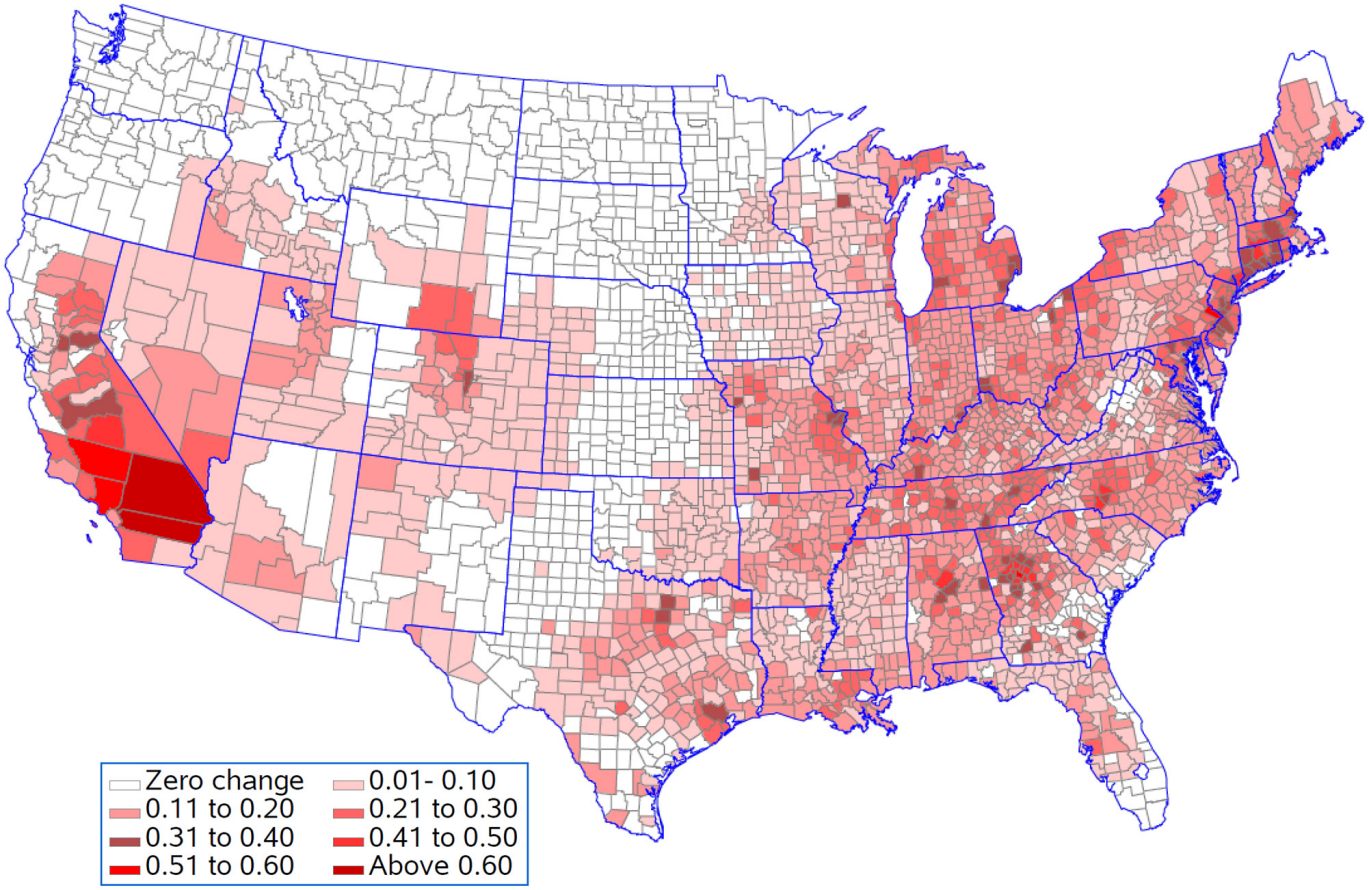
Reduction in life expectancy (years lost) resulting from being above the national 25^th^ percentile for each pollutant.

**Fig 8:**
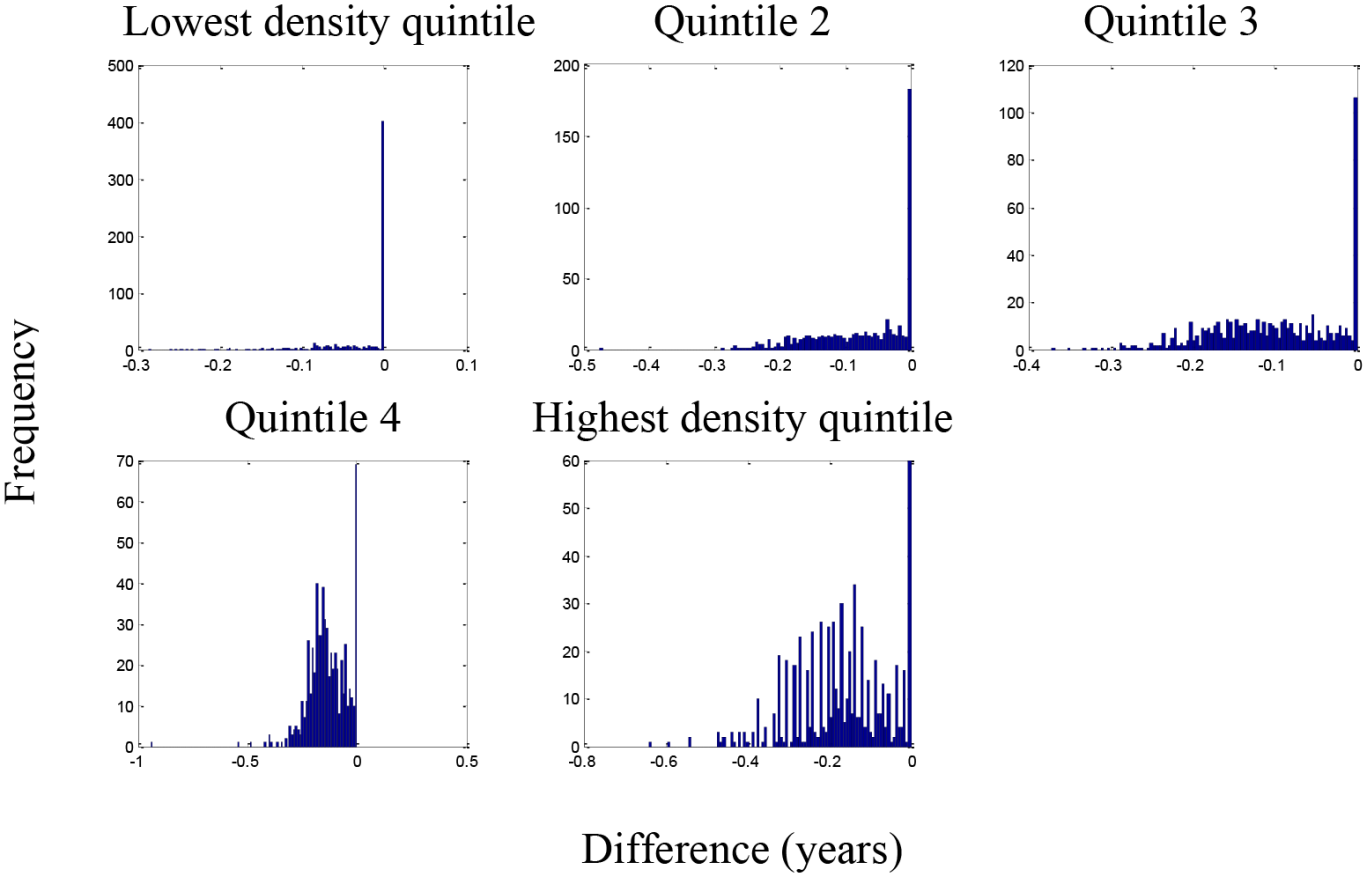
Change in life expectancy (years lost) resulting from being above the national 25^th^ percentile for each pollutant.

**Fig 9:**
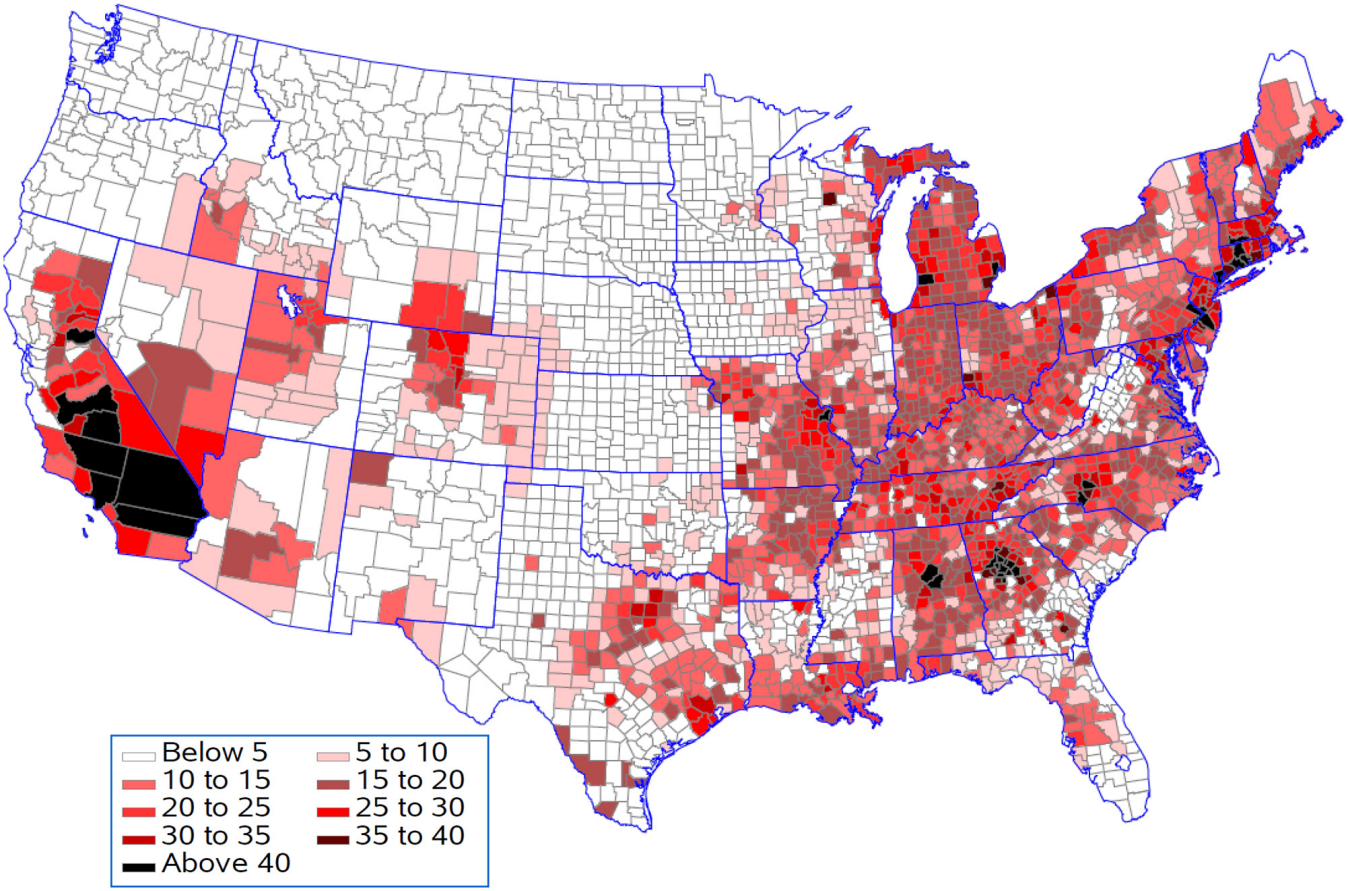
Increase in death from death from all causes (per 100,000 population per year) resulting from being above the national 25^th^ percentile for each pollutant.

**Fig 10:**
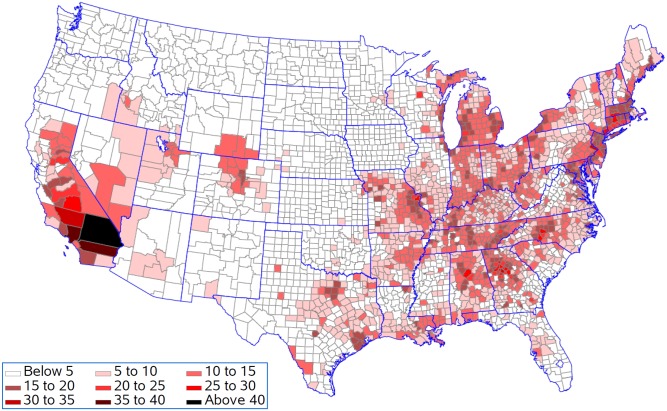
Increase in death from cardiovascular diseases (per 100,000 population per year) resulting from being above the national 25^th^ percentile for each pollutant.

Fig 7 shows that life expectancy is associated with being above the national 25^th^ percentile for ozone as predicted by the resulting regression model for five population density groups. This analysis presumes that all counties should be able to achieve reductions in pollution levels that could drop them down to this level. Life expectancy for 397 of the 622 counties remained unchanged in the lowest population density group with an average reduction in life expectancy of 0.08 years in the 225 counties with changes (Fig 8). In contrast, counties in the highest population density group had 568 out of 622 counties changed with an average reduction in life expectancy of 0.19 years. Figs 9 and 10 present the similar type of patterns for all-causes mortality and cardiovascular diseases. S7–S10 Figs present the similar type of patterns for combination of cardiovascular diseases, cancers and COPD; cancers and; COPD respectively for five population density groups. The mortality rate of COPD remained unchanged due to no pollutants entering into the regression model.

**Fig 11:**
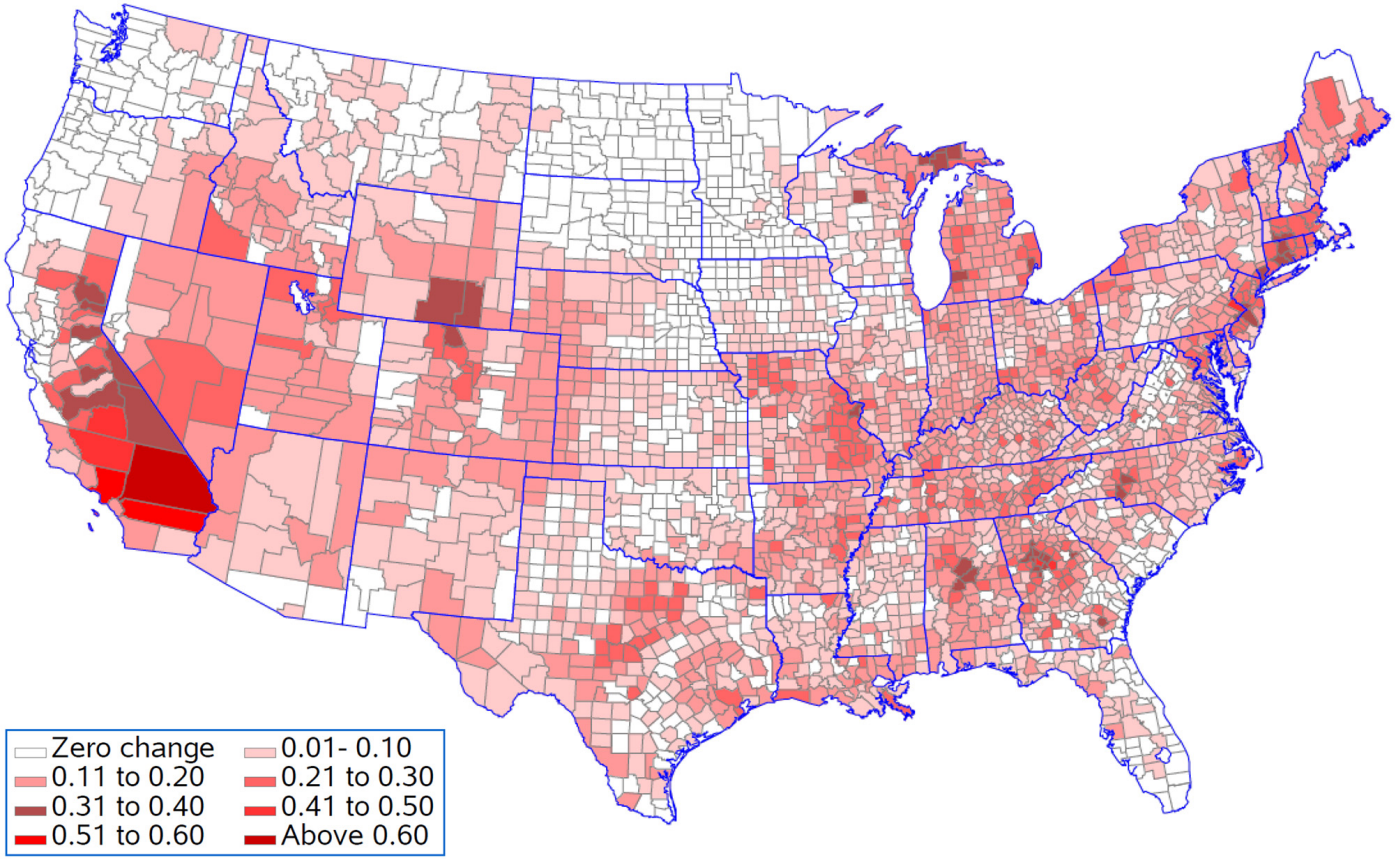
Reduction in life expectancy (years lost) resulting from being above the regional 25^th^ percentile for each pollutant.

**Fig 12:**
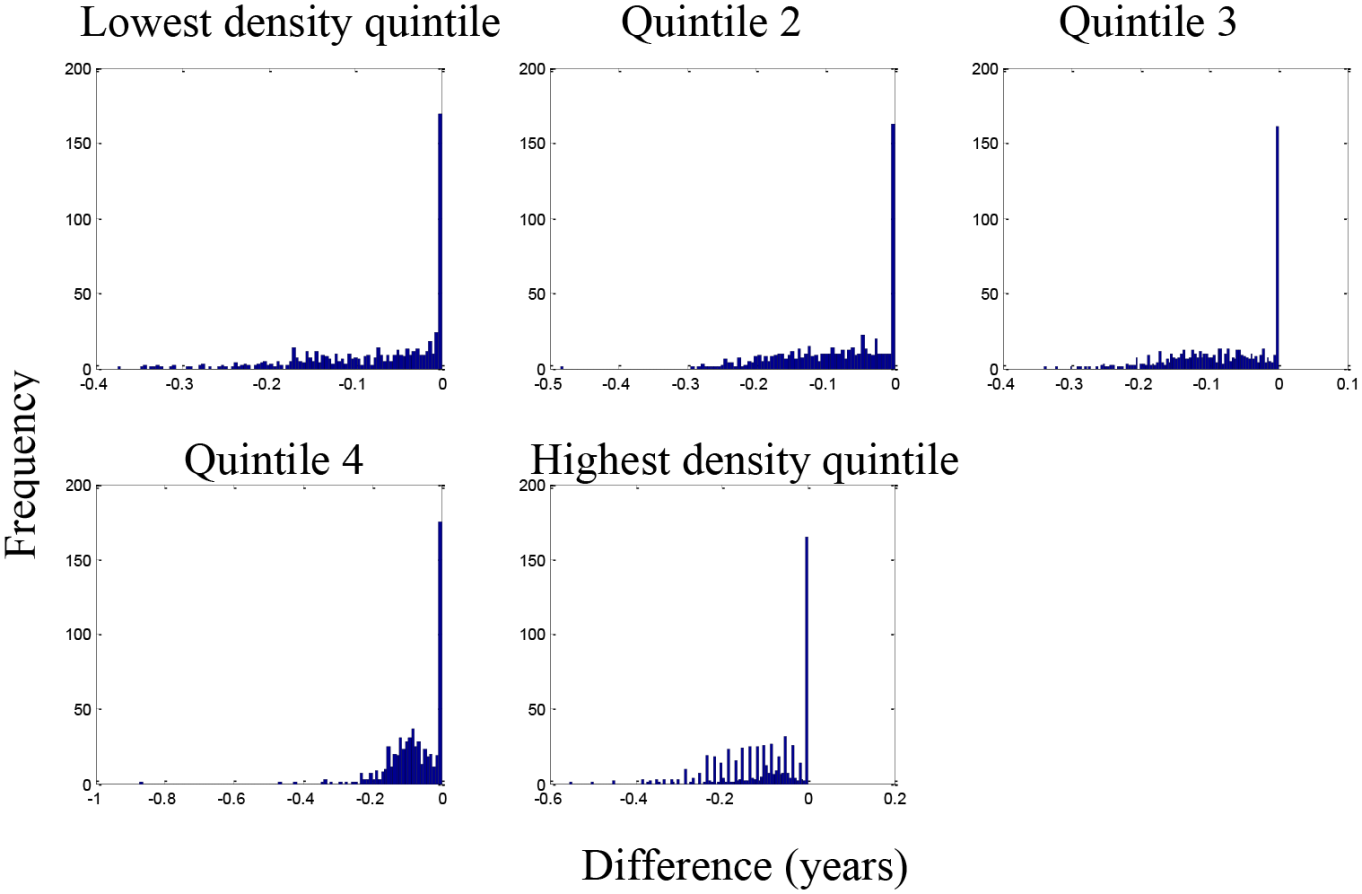
Change in life expectancy (years lost) resulting from being above the regional 25^th^ percentile for each pollutant.

**Fig 13:**
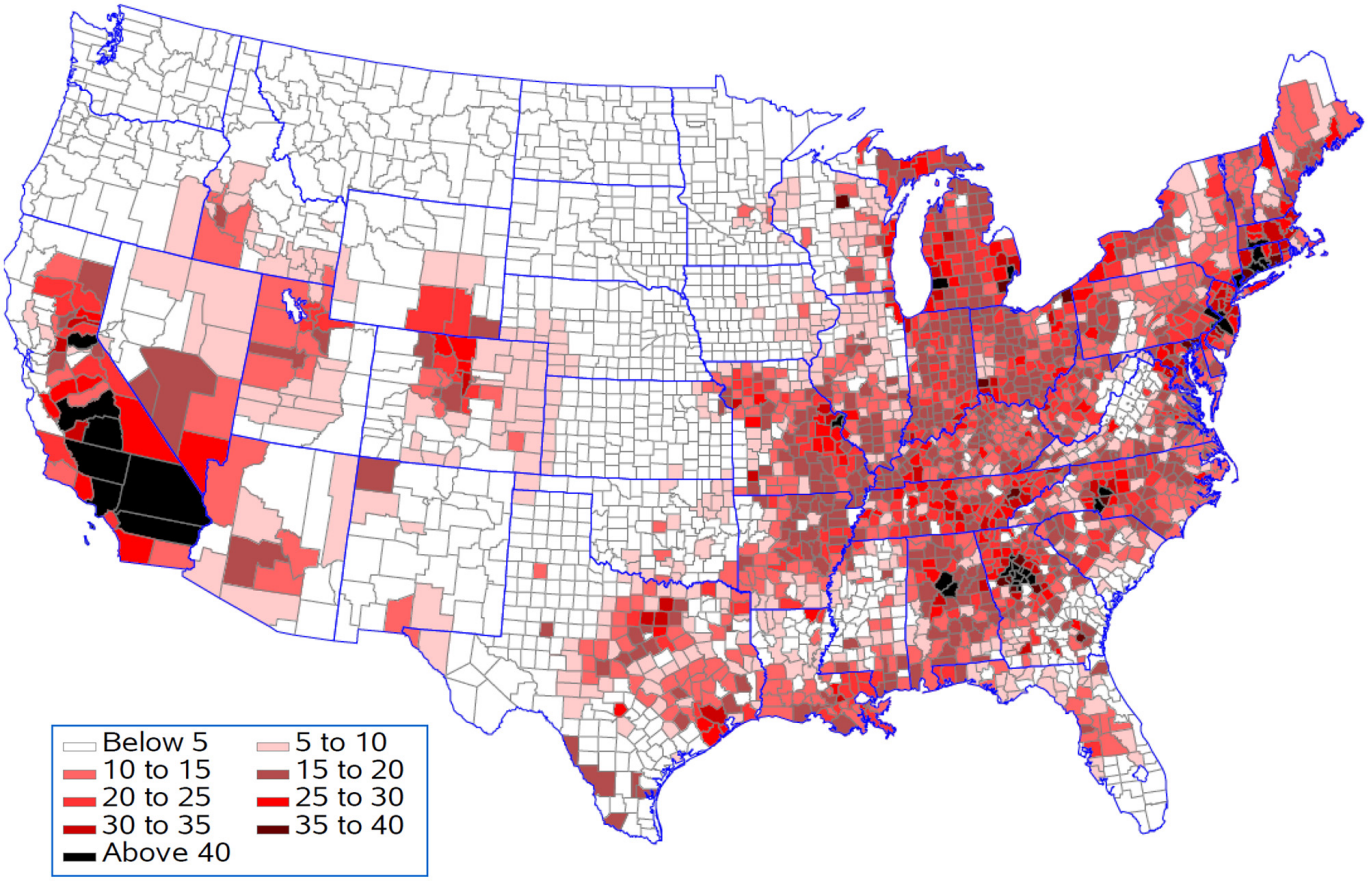
Increase in death from all causes (per 100,000 population per year) resulting from being above the regional 25^th^ percentile for each pollutant.

**Fig 14:**
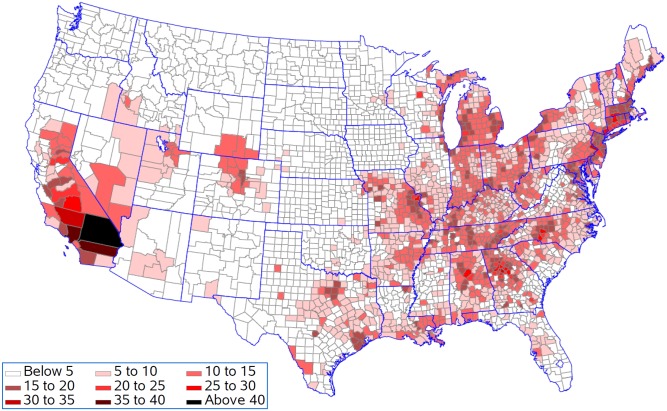
Increase in death from cardiovascular diseases (per 100,000 population per year) resulting from being above the regional 25^th^ percentile for each pollutant.

Fig 11 shows the reduction in life expectancy resulting from being above the 25^th^ percentile for ozone for counties in the five population density groups. This analysis is appropriate if population density sets limits for how much reduction in ozone is practical. In each population density group, 75% of the counties would see improvements in life expectancy (Fig 12). The improvements were 0.10, 0.11, 0.11, 0.10, and 0.13 years in the smallest to largest population density groups respectfully. Figs 13 and 14 present the similar type of patterns for all-causes mortality and cardiovascular diseases.

Generally counties in the Southern California had the biggest years lost in life expectancy as compared to counties in other states. Riverside County and San Bernardino County had 0.64 and 0.94 years lost in life expectancy when ozone was reduced to the national 25^th^ percentile and 0.55 and 0.87 years lost in life expectancy when all environmental pollutants were reduced to the regional 25^th^ percentile.

S7 and S8 Figs present an increase in death from combination of cardiovascular diseases, cancers and COPD (per 100,000 population per year), and cancers (per 100,000 population per year) resulting from being above the national 25^th^ percentile for each pollutant.

S9 and S10 Figs present an increase in death from combination of cardiovascular diseases, cancers and COPD (per 100,000 population per year), and cancers (per 100,000 population per year) resulting from being above the regional 25^th^ percentile for each pollutant.

## Discussion

Our findings found that Southern California, the Atlanta area, the Charlotte area, Birmingham, Bucks County, Pennsylvania, Hampden County, Massachusetts, Westchester County, New York, Philadelphia County, Pennsylvania and the adjacent Hartford County, Connecticut had the greatest changes in life expectancy when ozone was reduced to the national 25^th^ percentile under the assumption that counties should be able to achieve reductions in pollution levels that could drop them down to this level. The eastern region, central and southern California and the western region of Oregon and Washington have the highest concentrations for most of the air pollutants although the combined concentration of all pollutants is highest in the lower Midwest, the inland Southeast and the mid-Atlantic. The western region of the country has low volumes of pollutants, except for southern California. One of the major causes of the relative unhealthiness in the US population is due to areas in the Southeast which have high regional pollution caused by emissions from power plants, transportation, and/or extensive heavy industries [59]. Studies have indicated that people with limited access to resources have increased responses to air pollution, and there have been some correlations between socio-economic status (SES), particulate matter exposure, and mortality [60–63].

Counties in the Southern California generally had the biggest years lost in life expectancy as compared to counties in other states when all environmental pollutants were reduced to the regional 25^th^ percentile. In the Los Angeles area, the Great Basin is almost completely enclosed by mountains on the north and east. The vertical temperature structure (inversion) tends to prevent vertical mixing of the air through more than a shallow layer (1,000 to 2,000 feet deep). The geographical configuration and the southerly location of the Great Basin permit a fairly regular daily reversal of wind direction-offshore at night and onshore during the day. It is known that the annual prevailing wind direction in this region is West-North West (WNW). With the concentrated population and industry, pollution products tend to accumulate and remain within this circulation pattern, therefore affecting survival and life expectancy in those counties. We also found that only for cardiovascular diseases did we see a contribution of particulate matter to the regression model. Brunekreef suggested that the effect of long term exposure to low concentrations of fine particulate matter in air may also lead to a reduction of life expectancy of more than a year [64]. Recent reviews of literature demonstrated a significant increase in the risk of death from cardiovascular causes in association with an increase in ozone concentration and the risk of dying from a respiratory cause was found to be three times greater in the metropolitan areas with the highest concentrations as compared to those with the lowest concentrations. These studies also suggest that particulate matter has a primary role in adverse health effects on cardiopulmonary disease and death [65, 68].

In this study, we found that ozone, which is one of the most toxic photochemical pollutants, entered into our regression models for mortality due to all-causes; combination of cardiovascular diseases, cancers and COPD; cardiovascular diseases, cancers and for reductions in life expectancy. However, ozone did not enter into our regression model for mortality due to COPD and its mortality rate remained unchanged. Other environmental variables except ozone had no impact on the quality of the fit when they were removed from the analysis [65–67]. It is generally well known that higher temperatures and higher ozone are often correlated. It has been shown that the interaction between temperature and ozone was not significant when effect modification was assessed by temperature [69]. Social variables of interest are also quite likely to be correlated with each other and sometimes with environmental exposures, depending on the county or community. Therefore, making inference about their independent effects may be difficult if not impossible. Nevertheless, the results in our study suggests that it may be important to mitigate ozone exposure as it contributes to significant and measurable improvements in human health and life expectancy in the US. An important caveat that needs to be taken into account with the present study is that the data used in this study were assembled from available data sets from different sources, not studies designed specifically for the present study. In addition, ozone data were not available for all counties because according to the US EPA, ozone is not measured in counties known to be in compliance (that is, with low values). Therefore, the ozone data in the US EPA tables may be biased towards counties with high concentrations.

Since health disparities in the US have long been the subject of extensive scrutiny and analysis by both governmental and privately-funded organizations, numerous investigations have documented the findings in all measures of mortality by environmental hazards, climate, socioeconomic status and social capital [70–71]. Analyses using county-level and race/ethnic-specific mortality data have shown substantial variation across localities, some of which is related to socioeconomic levels [71]. We found that several other variables such as population being single parent or Hispanic or Latino, counties with high percentage of adults reporting no exercise or high blood pressure, percentage of males or females with at least a bachelor degree, religious organizations per 10,000 population, social organizations per 10,000 population, percentage of votes cast for President in a community, and total suicide death per 100,000 population entered into most regression models for many of the population density groups. Increases in mortality and decreases in life expectancy were seen for counties with a high percentage of adults reporting no exercise. This is not surprising as these variables are known to determine health-related quality of life and affect longevity directly [72]. This is also consistent with the Healthy People 2010 Report which indicated that a high percentage of the population being Hispanic or Latino increased life expectancy and reduced mortality from most of the causes studied [73]. Other factors such as differences in insurance coverage, access and utilization of care and quality of care have been investigated elsewhere. Substantial disparities that exist in mortality and functional health status within race/ethnic groups as a function of income, social class, education, and community deprivation have also drawn much attention [74–76]. Most metropolitan areas also tend to have a lower percentage of people with advanced education, and higher percentage of people in poverty. However, the groups with higher mortality do not have worse levels of all identifiable risk factors, nor do they have worse access to general health care as measured in the CDC’s Behavioral Risk Factor Surveillance System (BRFSS). Several investigations on racial residential segregation in the US reported some of the worse health outcomes among residents of racially segregated areas [77–79].

Social capital may have an important environmental influence, although it is not the sole determinant of increasing mortality rates. A study indicated that social engagement appeared to have modest protective effect on cardiovascular disease mortality independent of behavioral factors, socioeconomic conditions, disease, and disability in older men. The risk of lung cancer mortality also decreased among populations living in high social capital neighborhoods [80]. Skrabski et al. [81] indicated that mortality rates were closely related with levels of mistrust and social capital variables of the opposite sex seemed to have a protective effect for the other sex. Ethnic heterogeneity within the neighborhood might also play an important role in influencing the relation between social capital and mortality. In our regression model with five population density groups, five outlying counties were consistently observed in the lowest population density group and those counties were Bernett, Jackson, Mellette, Todd and Shannon, all of which were from South Dakota. These counties are some of the poorest in the nation and contain the Pine Ridge Indian Reservation, the poorest Indian reservation in the nation with severe unemployment (43% of those over 16) and with 49% of the people below the Federal poverty level as reported by the US Census Bureau. Removing these counties had virtually no impact on the quality of the fit in our analyses.

Cautions should be taken when interpreting the results as some of the variables used in the present study may act as potential confounders, surrogates or effect modifiers for other factors. In addition, interaction or modification effect was not taken into consideration in our study. For counties with missing data for a specific variable, data from the three closest counties regardless of population density were averaged to impute a value under the assumption that these counties demonstrate similar or almost similar characteristics with the three closest counties.

This analysis had several strengths. First, it is the first study, using large national data, to report and identify factors affecting the health outcomes of population based on county level data from across the US. More than 99% of the data were available for all counties for the variables that we used in the study except for ozone because according to the US EPA, ozone is not measured in counties known to be in compliance (that is, with low values). Second, the estimations of the models for all diseases as discussed in this study for the five population density groups were generally acceptable based on the R-squared values (R^2^>0.7) for almost all the plots. However, caution should be exercised as there is a slight lack-of-fit in the model, under-predicting the higher mortality rates and over predicting the lower mortality rates, suggesting some unmeasured or more fundamental factors missing from the linear predictor. Nevertheless, this study can be helpful to provide a basis for targeted control interventions and strategies as well as allocation of public health resources for county managers and authorities in a more cost-effective way.

This analysis also had several limitations. First, our study is an ecological study which relies on cross sectional data and cannot be used to assert cause and effect. Hence, when interpreting the results in this study, caution must be taken to avoid the potential for the ecological fallacy. Second, the study was conducted with only 3,110 of the 3,141 total counties in the US due to the paucity of reliable data in the remaining counties. Third, the variation of climate data on areas smaller than a county may affect the results due to the selection of weather station, geographic setting, cultural and socioeconomic influences and varying effects of different pollutant mixtures. Fourth, the utility of education, primary care and income as indicators of social class may be limited by the fact that their relationships with social class may have changed over time. These variables may also act as potential confounders or effect modifiers in the present study. It is unclear whether these variables or other variables used in this study contributed to the possible differences observed in mortality rates of different diseases over time. Fifth, because our study was population-based, we were limited in our ability to control geographic mobility and other additional potential confounders, especially various individual and community risk factors that may have been affected by policies that were broadly related to environmental regulation. Sixth, we used different data from different sources because not all data were available for the same year. The period of the data that we chose was as close to each other’s period as possible to minimize large difference. The difference of the data from one year to another year for any particular variable used in the model, if available, was less than 1%. Also, most of the variables that we used in the study are slow moving in the time dimension. Hence, it was concluded that the period of the data being used and any bias induced by the heterogeneity in sampling time, which is believed to be small, did not have any major impact in our analyses. Finally, the requirement that the effects of the air pollutants be detrimental and common across all population groups precluded modifying effects seen for some pollutants. When this restriction was removed and the individual population subgroups were allowed to have different pollutants drive the predictions, ozone remained as the pollutant which consistently entered in the model except for COPD, but other air pollution variables were seen to enter the regression for some population density groups. However, the effects were inconsistent and removing the restriction had virtually no impact on the quality of the fit. Therefore, we refrained from drawing strong causal relation between ozone or any variable and health outcomes due to the limitations in our approach. The results reported in the present study should be read with caution. Nevertheless, the results obtained from this research provide important policy implication. It is hoped that the results may further help us to improve the strength of the current models by considering approaches such as combining multiple collinear variables, interaction or effect modification, or other factors to completely characterize the health outcomes or health risks due to air pollution.

## Conclusions

Our study is the first to report and identify factors affecting the health outcomes of population based on county level data from across the US. Using new datasets and units of analysis, this study carries important policy implication and may provide prospective and additional impetus in the future to determine the health status of each county and provide a tool for county managers and authorities to use in evaluating the impact of changes they might make in their counties such as attracting more physicians, improving jobs and reducing environmental exposures. Since policies and resources aimed at reducing fundamental socioeconomic inequalities are limited in the US, understanding and quantifying the impacts of these inequalities should serve as a guide for addressing health disparities through public health reforms that reduce risk factors for chronic diseases and injuries. Although multiple factors affect survival and life expectancy, this study illustrates that a reduction in exposure to ozone contributes to significant and measurable improvements in human health and life expectancy in the US. However, the results reported in the present study should be read with caution.

As evidenced in the literature, it is generally well known that any differences in modeling parameters and approaches are likely to yield differences in health outcomes. Continued research is needed to improve data collection and develop more appropriate and concrete models including time-series analysis to be used to predict the mortality rate of a disease. Additional categories such as accidents, infectious diseases, infant mortality, occupational exposures, drinking water quality, allergens, public health interventions, unintentional injuries, drinking behaviors, and cross-county migration could be added to improve the strength of the current models.

## Supporting Information

S1 Fig Set 1: The estimated mortality plot for combination of cardiovascular diseases, cancers and COPD. Observed versus estimated mortality in 2,591 counties in the prediction set (Set 1) using stepwise regression for five population density groups (R-squared = 0.6494).
(PDF)

S2 Fig Set 2: The estimated mortality plot for combination of cardiovascular diseases, cancers and COPD. Observed versus estimated mortality in 519 counties in the validation set (Set 2) using stepwise regression for five population density groups.
(PDF)

S3 Fig Set 1: The estimated mortality plot for cancers. Observed versus estimated mortality in 2,591 counties in the prediction set (Set 1) using stepwise regression for five population density groups (R-squared = 0.4928).
(PDF)

S4 Fig Set 1: The estimated mortality plot for cancers. Observed versus estimated mortality in 519 counties in the validation set (Set 2) using stepwise regression for five population density groups.
(PDF)

S5 Fig Set 1: The estimated mortality plot for COPD. Observed versus estimated mortality in 2,591 counties in the prediction set (Set 1) using stepwise regression for five population density groups (R-squared = 0.3732).
(PDF)

S6 Fig Set 1: The estimated mortality plot for COPD. Observed versus estimated mortality in 519 counties in the validation set (Set 2) using stepwise regression for five population density groups.
(PDF)

S7 Fig Increase in death from combination of cardiovascular diseases, cancers and COPD (per 100,000 population per year) resulting from being above the national 25^th^ percentile for each pollutant. (PDF)

S8 Fig Increase in death from cancers (per 100,000 population per year) resulting from being above the national 25^th^ percentile for each pollutant. (PDF)

S9 Fig Increase in death from combination of cardiovascular diseases, cancers and COPD (per 100,000 population per year) resulting from being above the regional 25^th^ percentile for each pollutant. (PDF)

S10 Fig Increase in death from cancers (per 100,000 population per year) resulting from being above the regional 25^th^ percentile for each pollutant. (PDF)

S1 Table Average Volumes of Particulates and Gaseous Pollutants in Five Population Density Groups. Values are in average.
(PDF)

S2 Table Average Numbers of Different Organizations in Five Population Density Groups. Values are in average. Unit is in unit per 10,000 population.
(PDF)

S3 Table Demographic Characteristics in Five Population Density Groups. Values are in average.
(PDF)

S4 Table Race and Ethnicity and Wealth Characteristics in Five Population Density Groups. Values are in average.
(PDF)

S5 Table Crime, Housing and Occupation Characteristics in Five Population Density Groups. Values are in average.
(PDF)

S6 Table Weather characteristics in Five Population Density Groups. Values are in average.
(PDF)

S7 Table Risk Factors in Five Population Density Groups. Values are in average.
(PDF)

S8 Table Range of Diseases per 100,000 Population in Five Population Density Groups. Values are in average.
(PDF)

S9 Table Regression Parameters Derived from Stepwise Regression Analysis of Variables for Life Expectancy for Five Population Density Groups. (PDF)

S10 Table Regression Parameters Derived from Stepwise Regression Analysis of Variables for All-Causes Mortality for Five Population Density Groups. (PDF)

S11 Table Regression Parameters Derived from Stepwise Regression Analysis of Variables for Cardiovascular Diseases for Five Population Density Groups. (PDF)

S12 Table Regression Parameters Derived from Stepwise Regression Analysis of Variables for Combination of Cardiovascular Diseases, Cancers and COPD for Five Population Density Groups. (PDF)

S13 Table Regression Parameters Derived from Stepwise Regression Analysis of Variables for Cancers for Five Population Density Groups. (PDF)

S14 Table Regression Parameters Derived from Stepwise Regression Analysis of Variables for COPD for Five Population Density Groups. (PDF)
