# Correction: Environmental Predictors of US County Mortality Patterns on a National Basis

**DOI:** 10.1371/journal.pone.0146506

**Published:** 2016-01-04

**Authors:** The PLOS ONE Staff

There is an error in affiliation 2 for author Robert S. Weinhold. Affiliation 2 should be: Independent Researcher and Journalist, Tucson, AZ, 85736, United States of America.

The images for S1 Fig through S6 Fig are incorrect. Please see the correct Supporting Information below. The publisher apologizes for these errors.

## Supporting Information

S1 Fig Set 1: The estimated mortality plot for combination of cardiovascular diseases, cancers and COPD. Observed versus estimated mortality in 2,591 counties in the prediction set (Set 1) using stepwise regression for five population density groups (R-squared = 0.6494).
(PDF)

S2 Fig Set 2: The estimated mortality plot for combination of cardiovascular diseases, cancers and COPD. Observed versus estimated mortality in 519 counties in the validation set (Set 2) using stepwise regression for five population density groups.
(PDF)

S3 Fig Set 1: The estimated mortality plot for cancers. Observed versus estimated mortality in 2,591 counties in the prediction set (Set 1) using stepwise regression for five population density groups (R-squared = 0.4928).
(PDF)

S4 Fig Set 1: The estimated mortality plot for cancers. Observed versus estimated mortality in 519 counties in the validation set (Set 2) using stepwise regression for five population density groups.
(PDF)

S5 Fig Set 1: The estimated mortality plot for COPD. Observed versus estimated mortality in 2,591 counties in the prediction set (Set 1) using stepwise regression for five population density groups (R-squared = 0.3732).
(PDF)

S6 Fig Set 1: The estimated mortality plot for COPD. Observed versus estimated mortality in 519 counties in the validation set (Set 2) using stepwise regression for five population density groups.
(PDF)
