# Supplementary figures and images for: Environmental Predictors of US County Mortality Patterns on a National Basis
Source: PLoS One. 2015 Dec 2;10(12):e0137832. doi: 10.1371/journal.pone.0137832 (PMC4668104; doi:10.1371/journal.pone.0137832)

**S1 Fig.**

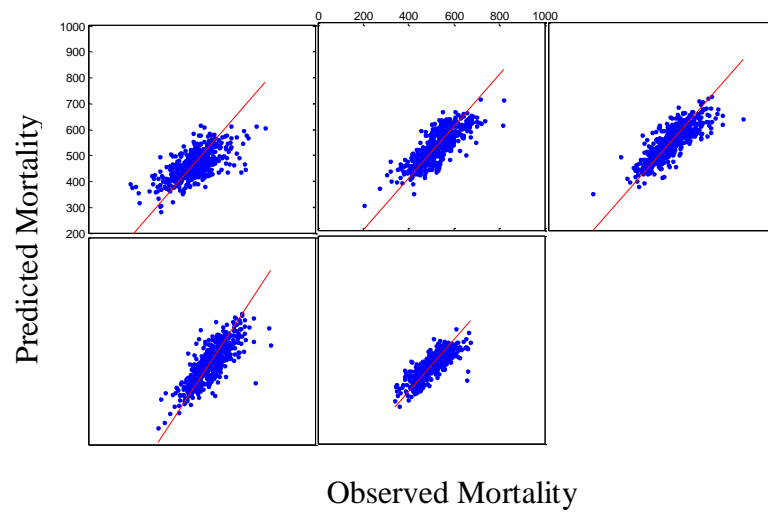

Supplement: S1 Fig — Observed versus estimated mortality in 2,591 counties in the prediction set (Set 1) using stepwise regression for five population density groups (R-squared = 0.6494). (PDF) [file pone.0137832.s001.pdf]

**S2 Fig.**

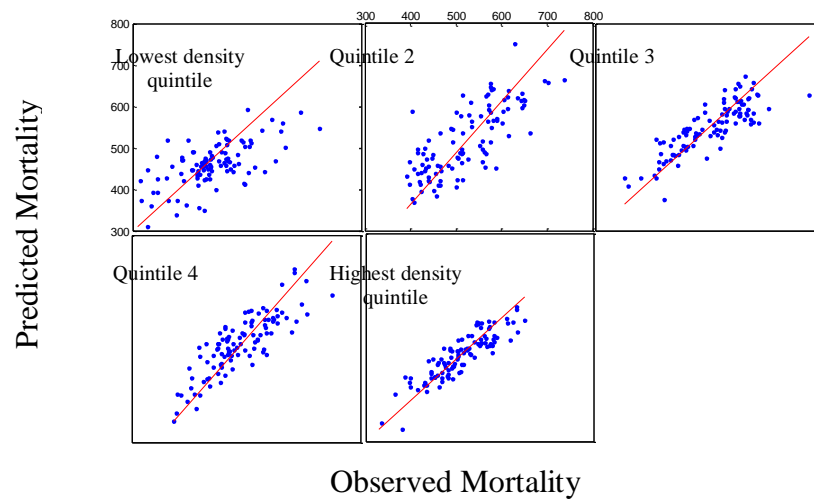

Supplement: S2 Fig — Observed versus estimated mortality in 519 counties in the validation set (Set 2) using stepwise regression for five population density groups. (PDF) [file pone.0137832.s002.pdf]

**S3 Fig.**

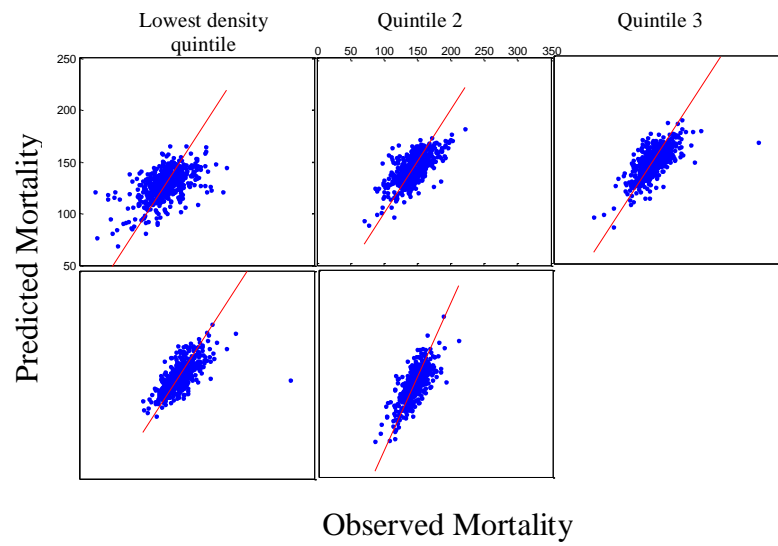

Supplement: S3 Fig — Observed versus estimated mortality in 2,591 counties in the prediction set (Set 1) using stepwise regression for five population density groups (R-squared = 0.4928). (PDF) [file pone.0137832.s003.pdf]

**S4 Fig.**

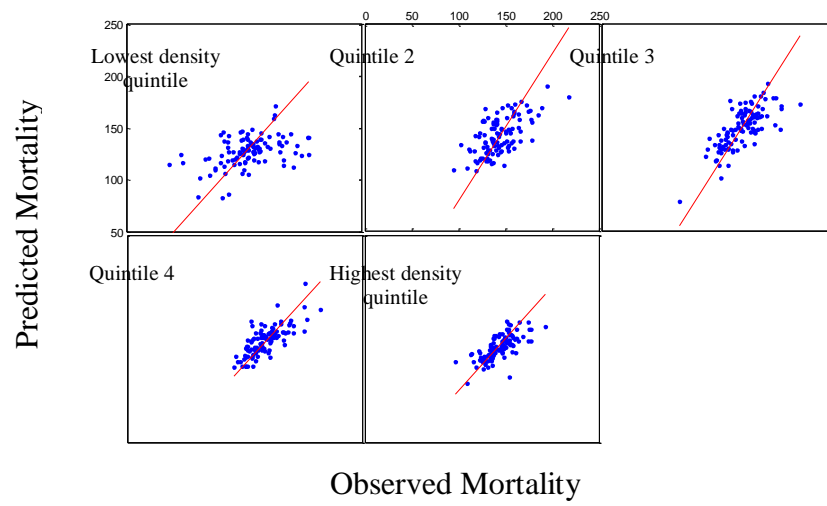

Supplement: S4 Fig — Observed versus estimated mortality in 519 counties in the validation set (Set 2) using stepwise regression for five population density groups. (PDF) [file pone.0137832.s004.pdf]

**S5 Fig.**

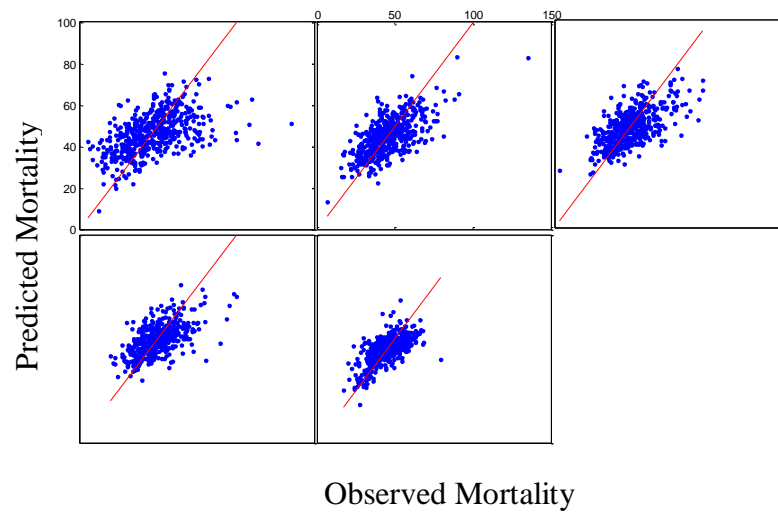

Supplement: S5 Fig — Observed versus estimated mortality in 2,591 counties in the prediction set (Set 1) using stepwise regression for five population density groups (R-squared = 0.3732). (PDF) [file pone.0137832.s005.pdf]

**S6 Fig.**

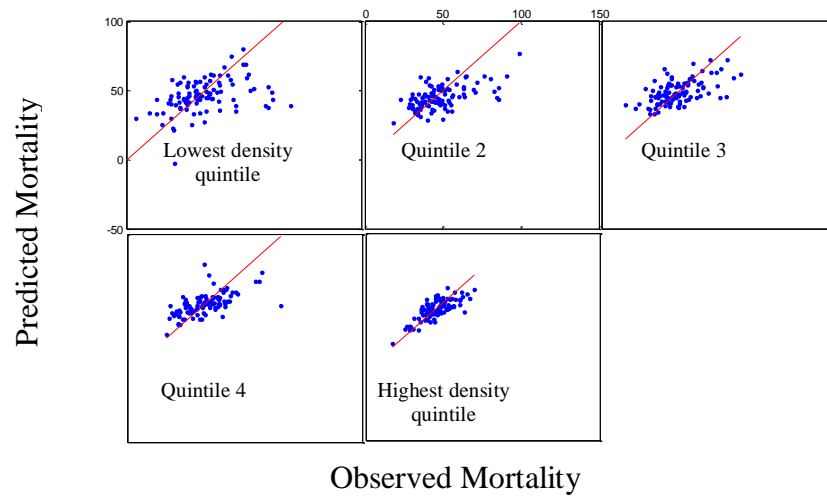

Supplement: S6 Fig — Observed versus estimated mortality in 519 counties in the validation set (Set 2) using stepwise regression for five population density groups. (PDF) [file pone.0137832.s006.pdf]

S7 Fig.

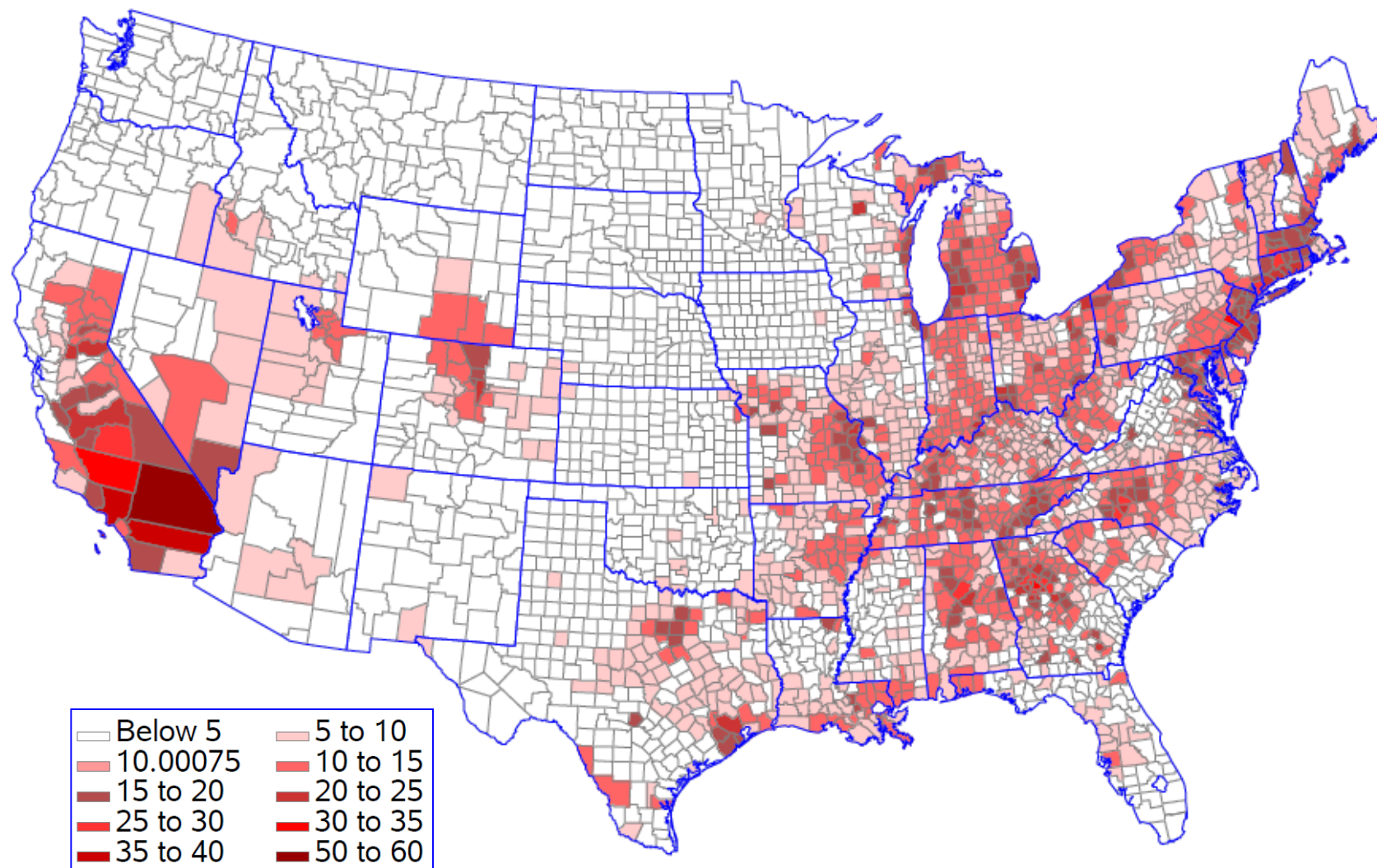

Supplement: S7 Fig — (PDF) [file pone.0137832.s007.pdf]

S8 Fig.

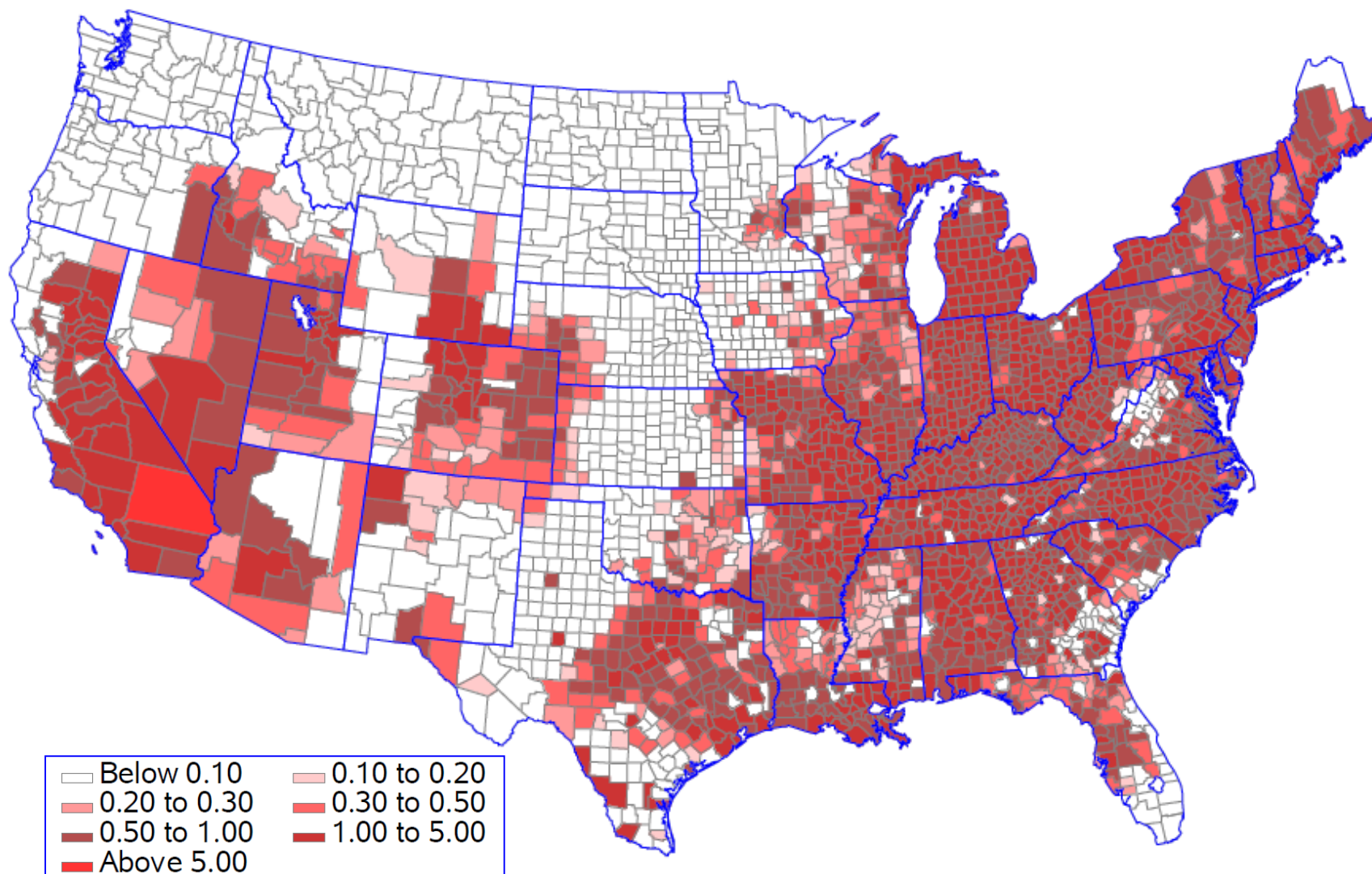

Supplement: S8 Fig — (PDF) [file pone.0137832.s008.pdf]

**S9 Fig.**

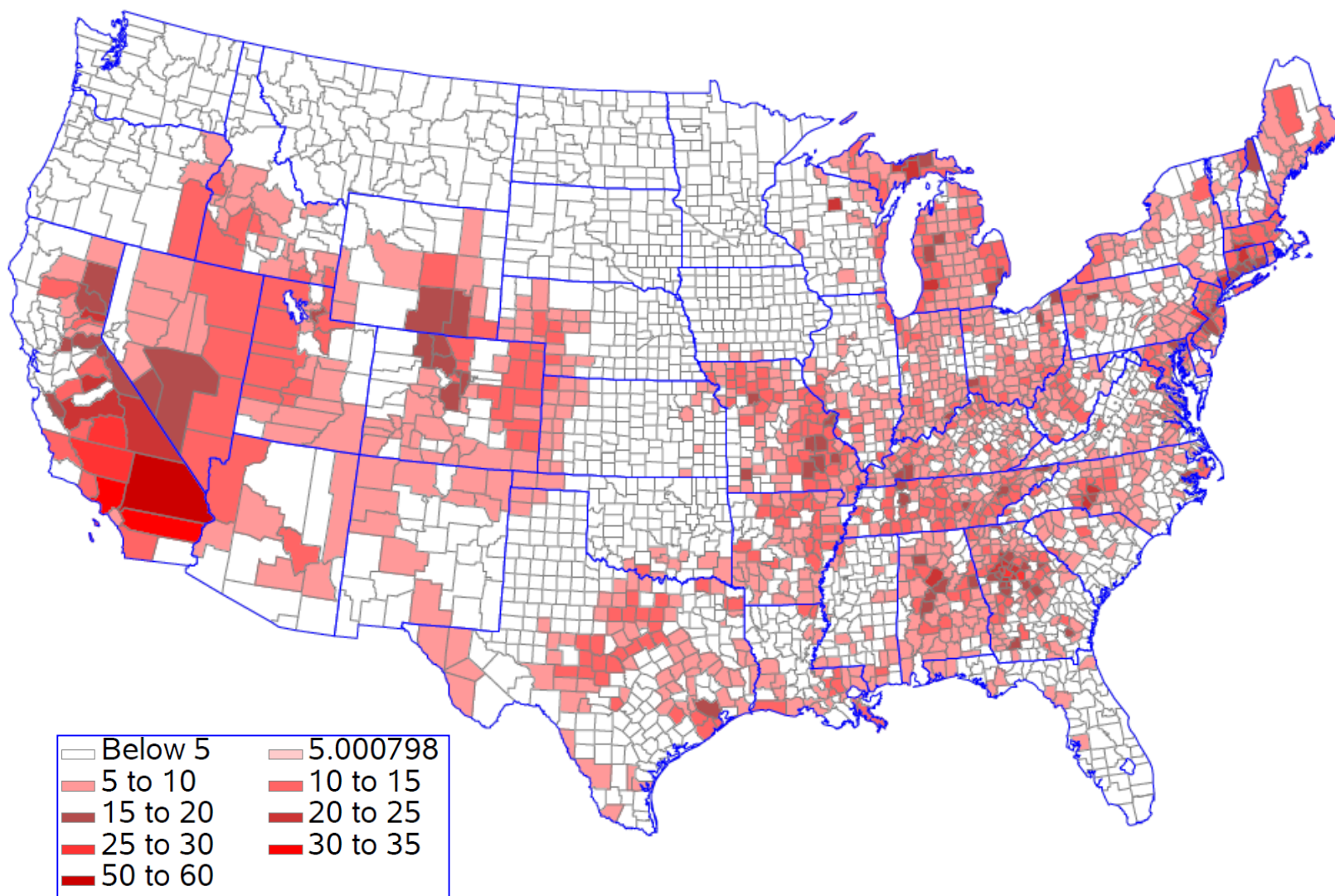

Supplement: S9 Fig — (PDF) [file pone.0137832.s009.pdf]

S10 Fig.

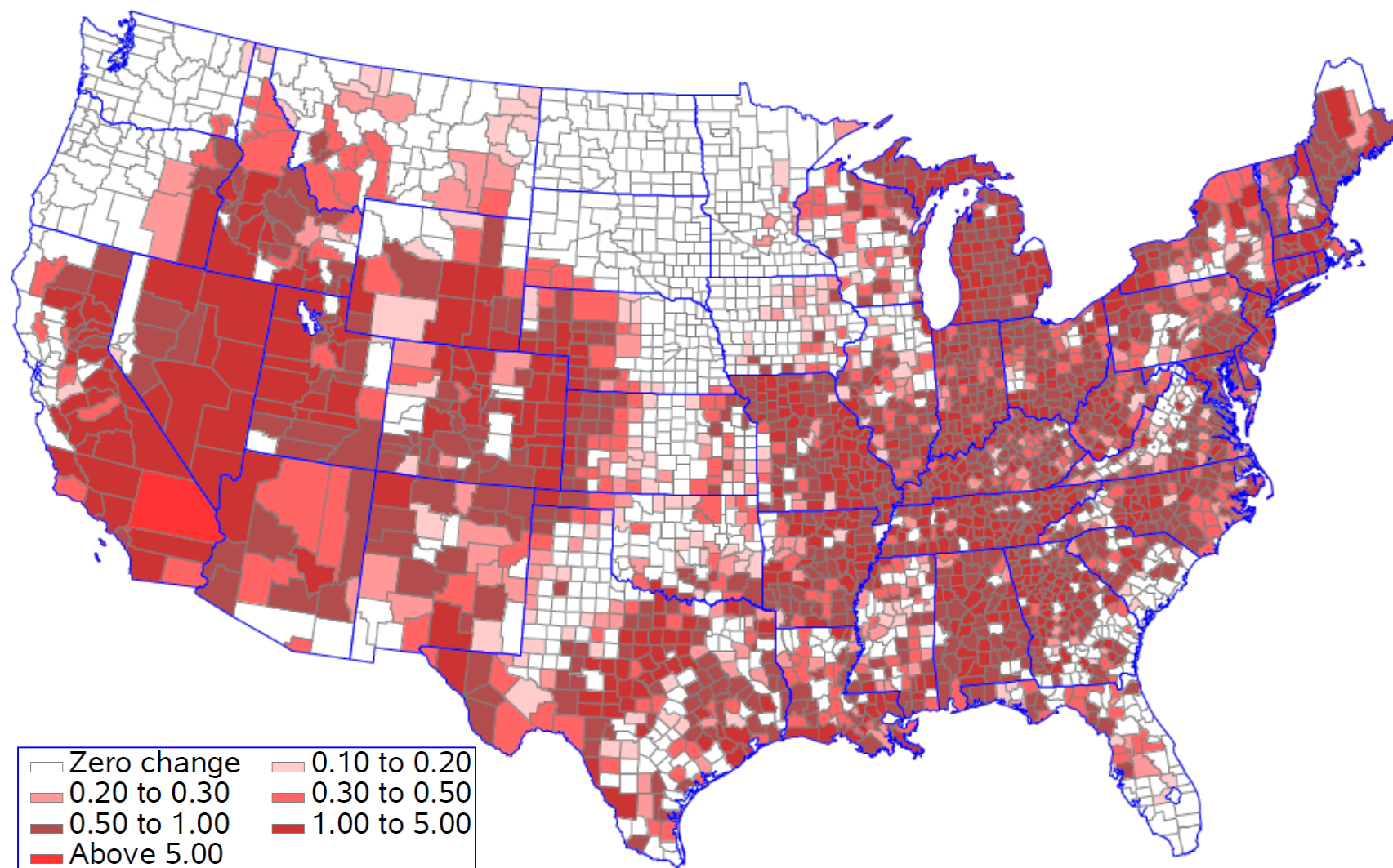

Supplement: S10 Fig — (PDF) [file pone.0137832.s010.pdf]
