# Supplementary material for: Environmental Predictors of US County Mortality Patterns on a National Basis
Source: PLoS One. 2015 Dec 2;10(12):e0137832. doi: 10.1371/journal.pone.0137832 (PMC4668104; doi:10.1371/journal.pone.0137832)
Supplement: S1 Table — Values are in average. (PDF) [file pone.0137832.s011.pdf]

S1 Table. Average Volumes of Particulates and Gaseous Pollutants in Five Population Density Groups. Values are in average.

	Quintile	Lowest density quintile	Quintile 2	Quintile 3	Quintile 4	Highest density quintile
	No. of Counties	622	622	622	622	622
Particulates	Diesel[1]	0.04	0.08	0.11	0.15	2.06
	PM_{10}	5.29	7.29	9.50	11.65	41.70
	$PM_{2.5}$	1.05	1.46	2.11	2.89	14.24
Gaseous pollutants	CO	12.20	21.36	37.66	61.64	472.90
	NH_3	1.15	1.85	1.95	2.37	5.89
	NO_x	2.86	4.90	9.00	13.53	106.16
	SO_2	1.25	2.88	8.28	13.38	110.06
	VOC	2.40	4.02	6.88	11.30	87.04
	O_3[2]	0.07	0.07	0.08	0.08	0.08

[1]Ton per square mile

[2]ppm
