# Supplementary material for: Environmental Predictors of US County Mortality Patterns on a National Basis
Source: PLoS One. 2015 Dec 2;10(12):e0137832. doi: 10.1371/journal.pone.0137832 (PMC4668104; doi:10.1371/journal.pone.0137832)
Supplement: S2 Table — Values are in average. Unit is in unit per 10,000 population. (PDF) [file pone.0137832.s012.pdf]

S2 Table. Average Numbers of Different Organizations in Five Population Density Groups. Values are in average. Unit is in unit per 10,000 population.

Quintile	Lowest density quintile	Quintile 2	Quintile 3	Quintile 4	Highest density quintile
No. of Counties	622	622	622	622	622
Bowling centers	0.65	0.36	0.27	0.26	0.21
Civic and social organizations	2.22	1.48	1.34	1.52	1.35
Physical fitness facilities	0.23	0.22	0.26	0.33	0.52
Public golf courses	0.62	0.41	0.33	0.39	0.18
Religious organizations	10.71	10.07	8.95	8.52	6.66
Sports clubs, managers and promoters	0.05	0.03	0.03	0.03	0.08
Memberships in sports and recreation clubs	0.98	0.80	0.63	0.61	0.63
Political organizations	0.01	0.02	0.02	0.03	0.10
Professional organizations	0.14	0.12	0.10	0.14	0.45
Business organizations	0.93	0.65	0.54	0.46	0.66
Labor organizations	0.20	0.33	0.57	0.69	0.83
Memberships in organizations not elsewhere classified	0.92	0.62	0.48	0.41	0.27
Aggregate for all of above variables	17.66	15.13	13.53	13.40	11.96
Non-profit organizations	4.14	3.35	3.29	3.68	6.43
Response rate (mail in) from the Census	59.07	61.25	61.40	63.70	68.14
% Votes cast for President	63.07	56.19	52.40	51.06	51.89
