# Supplementary material for: Environmental Predictors of US County Mortality Patterns on a National Basis
Source: PLoS One. 2015 Dec 2;10(12):e0137832. doi: 10.1371/journal.pone.0137832 (PMC4668104; doi:10.1371/journal.pone.0137832)
Supplement: S3 Table — Values are in average. (PDF) [file pone.0137832.s013.pdf]

S3 Table. Demographic Characteristics in Five Population Density Groups. Values are in average.

Quintile	Lowest density quintile	Quintile 2	Quintile 3	Quintile 4	Highest density quintile
No. of Counties	622	622	622	622	622
Population density (Maximum/square mile)	12.85	31.74	58.63	144.41	66940.07
% Rural population	80.46	73.28	68.48	55.98	6.87
% Foreign-born population	3.30	2.39	2.39	2.67	9.57
% Single parent households	8.02	9.81	10.08	10.01	10.83
% Married-Couple families	58.12	55.94	56.21	56.48	49.74
% 16-64 years old with physical disability (Both sexes)	1.30	1.45	1.52	1.46	1.17
% 16-64 years old with mental disability (Both sexes)	0.48	0.54	0.60	0.61	0.63
% 16-64 years old with employment disability (Both sexes)	2.94	3.12	3.30	3.32	3.23
% ≥65 years old with physical disability (Both sexes)	1.67	1.64	1.50	1.34	1.13
% ≥65 years old with mental disability (Both sexes)	0.19	0.17	0.16	0.15	0.12
% Males with at least a bachelor degree	15.94	13.16	13.48	16.27	30.53
% Females with at least a bachelor degree	15.64	13.28	13.42	15.27	26.20
Median age (Both sexes)	39.43	38.06	37.30	36.44	35.38
Doctors per 10,000 population	4.47	4.09	4.90	5.77	11.70
Dentists per 10,000 population	2.73	2.64	2.77	3.03	6.03
% Uninsured (All ages)	17.91	15.54	14.73	13.24	12.50
% People unemployed	4.58	6.01	6.44	6.24	5.55
