# Supplementary material for: Environmental Predictors of US County Mortality Patterns on a National Basis
Source: PLoS One. 2015 Dec 2;10(12):e0137832. doi: 10.1371/journal.pone.0137832 (PMC4668104; doi:10.1371/journal.pone.0137832)
Supplement: S4 Table — Values are in average. (PDF) [file pone.0137832.s014.pdf]

S4 Table. Race and Ethnicity and Wealth Characteristics in Five Population Density Groups. Values are in average.

	Quintile	Lowest density quintile	Quintile 2	Quintile 3	Quintile 4	Highest density quintile
	No. of Counties	622	622	622	622	622
Race and Ethnicity	% American Indian and Alaska Native	4.14	1.75	1.05	0.71	0.42
	% Asian	0.28	0.32	0.40	0.67	3.37
	% Black or African American	1.60	11.04	10.69	8.74	15.67
	% Hispanic or Latino	10.77	5.27	4.48	4.10	8.43
	% Native Hawaiian and Pacific Islander	0.04	0.04	0.03	0.04	0.09
	% White	88.08	83.34	84.85	86.78	74.74
	% Some other race	4.35	2.26	1.80	1.83	3.56
	% Two or more races	1.52	1.25	1.18	1.23	2.16
Wealth	% People below poverty line	15.55	15.85	15.55	13.00	10.91
	Median family income	31209.43	31405.55	32751.48	36698.91	46918.36
	Median household income	37364.64	37734.08	39091.48	43383.21	55947.90
	Per capita income (Total population)	15888.09	15793.88	16277.06	17774.36	23510.84
