# Supplementary material for: Environmental Predictors of US County Mortality Patterns on a National Basis
Source: PLoS One. 2015 Dec 2;10(12):e0137832. doi: 10.1371/journal.pone.0137832 (PMC4668104; doi:10.1371/journal.pone.0137832)
Supplement: S5 Table — Values are in average. (PDF) [file pone.0137832.s015.pdf]

S5 Table. Crime, Housing and Occupation Characteristics in Five Population Density Groups. Values are in average.

	Quintile	Lowest density quintile	Quintile 2	Quintile 3	Quintile 4	Highest density quintile
	No. of Counties	622	622	622	622	622
Crime	Murder per 100,000 population	1.73	2.90	2.81	2.71	6.31
	Assault per 100,000 population	125.53	148.50	165.97	185.12	281.79
	Total suicide death per 100,000 population	15.22	13.34	13.13	12.29	10.32
Housing	% Occupied housing units of total housing	78.35	83.98	85.72	88.84	93.57
	% Occupied housing units lacking full plumbing	1.07	0.97	0.84	0.62	0.29
	% Owner-Renter occupied housing units lacking plumbing	2.14	2.53	2.30	1.71	1.00
Occupation	% Employed in mining, construction, manufacturing, etc.	1013.36	1361.98	1471.05	1532.63	1195.08
	% Employed in agriculture, fishing, hunting, etc.	681.86	306.92	189.52	122.76	20.71
