# Supplementary material for: Environmental Predictors of US County Mortality Patterns on a National Basis
Source: PLoS One. 2015 Dec 2;10(12):e0137832. doi: 10.1371/journal.pone.0137832 (PMC4668104; doi:10.1371/journal.pone.0137832)
Supplement: S6 Table — Values are in average. (PDF) [file pone.0137832.s016.pdf]

S6 Table. Weather characteristics in Five Population Density Groups. Values are in average.

Quintile	Lowest density quintile	Quintile 2	Quintile 3	Quintile 4	Highest density quintile
No. of Counties	622	622	622	622	622
Maximum temperature in January	41.8	43.1	41.1	39.7	42.9
Maximum temperature in August	86.1	86.3	85.2	85.0	86.5
Minimum temperature in January	20.9	21.2	19.5	18.1	21.9
Minimum temperature in August	61.3	60.2	58.3	58.1	61.2
Average temperature in January	31.4	32.1	30.3	28.9	32.4
Average temperature in August	73.7	73.3	71.8	71.6	73.9
Hot degree day in January	34.6	34.8	35.3	34.6	32.7
Hot degree day in August	1058.8	1078.6	1067.0	1065.4	1029.2
Cold degree day in January	34.0	30.8	30.9	31.6	32.8
Cold degree day in August	0.9	0.5	1.0	0.7	1.0
Average annual precipitation	256.1	271.4	286.1	265.4	269.9
