# Supplementary material for: Environmental Predictors of US County Mortality Patterns on a National Basis
Source: PLoS One. 2015 Dec 2;10(12):e0137832. doi: 10.1371/journal.pone.0137832 (PMC4668104; doi:10.1371/journal.pone.0137832)
Supplement: S7 Table — Values are in average. (PDF) [file pone.0137832.s017.pdf]

**S7 Table. Risk Factors in Five Population Density Groups. Values are in average.**

| Quintile | Lowest density quintile | Quintile 2 | Quintile 3 | Quintile 4 | Highest density quintile |
|---|---|---|---|---|---|
| No. of Counties | 622 | 622 | 622 | 622 | 622 |
| % Adults reporting no exercise | 26.25 | 27.49 | 28.70 | 27.59 | 23.61 |
| % Adults reporting an average of fruit and vegetables consumption of less than 5 servings per day | 77.97 | 78.66 | 77.20 | 77.00 | 74.68 |
| % Adults who are obese | 25.09 | 26.56 | 26.70 | 25.31 | 21.79 |
| % Adults reporting high blood pressure | 26.16 | 28.91 | 29.38 | 28.39 | 25.06 |
| % Smokers | 20.06 | 22.73 | 24.37 | 25.04 | 21.03 |
| % Adults reporting diabetes | 8.28 | 8.42 | 8.49 | 8.12 | 6.92 |
