# Supplementary material for: Environmental Predictors of US County Mortality Patterns on a National Basis
Source: PLoS One. 2015 Dec 2;10(12):e0137832. doi: 10.1371/journal.pone.0137832 (PMC4668104; doi:10.1371/journal.pone.0137832)
Supplement: S8 Table — Values are in average. (PDF) [file pone.0137832.s018.pdf]

S8 Table. Range of Diseases per 100,000 Population in Five Population Density Groups. Values are in average.

Quintile	Lowest density quintile	Quintile 2	Quintile 3	Quintile 4	Highest density quintile
No. of Counties	622	622	622	622	622
All causes mortality (i)	821.53	898.50	929.90	914.02	855.27
Cardiovascular diseases (ii)	292.55	336.40	348.48	338.93	310.78
Cancers (iii)	127.61	143.64	148.23	147.74	141.12
COPD (iv)	46.16	45.51	48.43	49.66	41.31
Combination of (ii)-(iv)	466.31	525.55	545.14	536.33	493.21
Life expectancy	76.98	76.09	75.66	76.05	76.97
