# Supplementary material for: Environmental Predictors of US County Mortality Patterns on a National Basis
Source: PLoS One. 2015 Dec 2;10(12):e0137832. doi: 10.1371/journal.pone.0137832 (PMC4668104; doi:10.1371/journal.pone.0137832)
Supplement: S9 Table — (PDF) [file pone.0137832.s019.pdf]

S9 Table. Regression Parameters Derived from Stepwise Regression Analysis of Variables for Life Expectancy for Five Population Density Groups.

Variable	Lowest Density Quintile			Quintile 2			Quintile 3			Quintile 4			Highest Density Quintile		
	Regression coefficient	Standard deviation	P value	Regression coefficient	Standard deviation	P value	Regression coefficient	Standard deviation	P value	Regression coefficient	Standard deviation	P value	Regression coefficient	Standard deviation	P value
Intercept Term							78.58	0.322	0						
Ozone							-0.1288	0.02404	9.20E-08						
% Foreign-born population										0.2913	0.09938	0.003404	0.2363	0.03519	2.34E-11
% Single parent households	-0.366	0.05238	3.55E-12	-0.2809	0.09693	0.003785	-0.6453	0.06914	0	-0.6837	0.07966	0			
% 16-64 years (Both sexes) with physical disability				-0.2238	0.04946	6.31E-06	-0.154	0.05502	0.005179	-0.2219	0.06478	0.000625	-0.8324	0.08994	0
% ≥65 years (Both sexes) with mental disability										0.221	0.06713	0.001007	0.3284	0.06507	4.81E-07
Civic and social organizations per 10,000 population	0.106	0.02695	8.58E-05	0.2408	0.05233	4.40E-06									
Aggregate for all of social capital variables per 10,000 population										-0.2028	0.07822	0.00958			
Memberships in sports and recreation clubs per 10,000 population	0.07192	0.02552	0.004863												
Business organizations per 10,000 population	0.09293	0.02526	0.000238												
Memberships in organizations not classified elsewhere per 10,000 population	-0.07926	0.02264	0.000471												
Non-profit organizations per 10,000 population							0.2324	0.07053	0.000997						
Response rate from the Census				0.3668	0.07273	4.90E-07				0.49	0.09941	8.78E-07	0.1344	0.06005	0.02531
% Votes cast for President							0.2218	0.06212	0.000363	0.4051	0.0561	6.82E-13	0.3019	0.06429	2.80E-06
% Males with at least a bachelor degree	0.2307	0.06712	0.000597				0.6022	0.09428	2.01E-10						
% Females with at least a bachelor degree				0.6239	0.08791	1.66E-12				0.3111	0.06563	2.25E-06			
Dentists per 10,000 population										0.2331	0.08205	0.00453			
% Uninsured (All ages)				-0.4508	0.08782	3.06E-07	-0.4592	0.1112	3.75E-05						
% People below poverty line	-0.3986	0.06163	1.19E-10				-0.3007	0.09252	0.001169	-0.3829	0.08134	2.66E-06			
% People unemployed				0.1581	0.04666	0.000713	0.1618	0.04888	0.000947						
Median household income				1.072	0.2847	0.00017							0.1821	0.05189	0.000459
Median family income				-1.043	0.3137	0.0009									
% Occupied housing units of total housing				-0.1686	0.07039	0.01666				-0.3223	0.09231	0.000489			
% Owner occupied housing units lacking plumbing							0.2233	0.09331	0.01677				1.025	0.2561	6.45E-05
% Black or African American	-0.47	0.1053	8.35E-06	-0.2732	0.07358	0.000209							-0.6594	0.06287	0
% Some other race	-0.2204	0.06732	0.001076				-0.3137	0.1198	0.008903						
% Two or more races	-0.336	0.0536	4.28E-10												
% Hispanic or Latino	0.4802	0.06525	2.49E-13	0.4485	0.06335	1.87E-12	1.019	0.1137	0	0.3268	0.09986	0.00108			
Maximum temperature in January	0.1786	0.04316	3.62E-05												
Cold degree day in January	-0.09746	0.03501	0.005409												
% Adults reporting no exercise				-0.3332	0.0512	9.14E-11	-0.2434	0.04958	9.75E-07	-0.1769	0.05927	0.002861	-0.2984	0.05916	4.88E-07
% Adults reporting high blood pressure	-0.2747	0.06334	1.50E-05	-0.1363	0.04689	0.003684	-0.1435	0.04974	0.003939	-0.136	0.04949	0.006039			
% Adults reporting an average of fruit and vegetables consumption of less than 5 servings per day	0.2063	0.04405	2.96E-06												
% Adults who are obese	-0.2862	0.04842	3.88E-09												
% Smokers													-0.327	0.06049	7.07E-08
% Adults reporting diabetes	0.0974	0.04135	0.01858												
Murder per 100,000 population							-0.1392	0.05662	0.01401	-0.2219	0.08093	0.006159	-0.2994	0.05622	1.10E-07
Total suicide death per 100,000 population							-0.1464	0.05673	0.009927	-0.2905	0.07842	0.000217			
People employed in mining, construction, manufacturing, etc. per 10,000 population				0.3974	0.09783	5.02E-05	0.8432	0.1345	4.26E-10						
