# Supplementary material for: Environmental Predictors of US County Mortality Patterns on a National Basis
Source: PLoS One. 2015 Dec 2;10(12):e0137832. doi: 10.1371/journal.pone.0137832 (PMC4668104; doi:10.1371/journal.pone.0137832)
Supplement: S10 Table — (PDF) [file pone.0137832.s020.pdf]

S10 Table. Regression Parameters Derived from Stepwise Regression Analysis of Variables for All-Causes Mortality for Five Population Density Groups.

Variable	Lowest Density Quintile			Quintile 2			Quintile 3			Quintile 4			Highest Density Quintile		
	Regression coefficient	Standard deviation	P value	Regression coefficient	Standard deviation	P value	Regression coefficient	Standard deviation	P value	Regression coefficient	Standard deviation	P value	Regression coefficient	Standard deviation	P value
Intercept Term							685.1	19.95	0						
Ozone							13.32	1.721	1.44E-14						
% Foreign-born population										-21.68	7.034	0.002072	-12.15	3.8	0.001402
% Single parent households	53.09	3.392	0	57.45	4.91	0	25.19	6	2.78E-05	38.74	4.997	1.31E-14	26.9	5.525	1.20E-06
% Married-Couple families				23.18	5.354	1.56E-05	-9.39	4.182	0.02482						
% 16-64 years (Both sexes) with physical disability	12.92	3.005	1.78E-05	17.36	3.628	1.80E-06	12.98	3.896	0.00088	18.52	4.623	6.34E-05	52.76	6.951	4.46E-14
% ≥65 years (Both sexes) with physical disability	9.533	2.854	0.000848												
% ≥65 years (Both sexes) with mental disability	-8.425	2.152	9.27E-05							-17.97	4.733	0.00015			
Religious organizations per 10,000 population	11.27	2.225	4.42E-07	9.31	3.936	0.01809							19.62	4.617	2.22E-05
Professional organizations per 10,000 population	-7.627	2.113	0.000313												
Response rate from the Census				-31.27	5.089	9.22E-10	-31.19	7.021	9.25E-06						
Labor organizations per 10,000 population										8.645	3.146	0.006045			
% Votes cast for President	-21.49	3.209	2.64E-11	-21.52	4.234	3.99E-07				-28.54	3.404	1.11E-16	-19.14	3.692	2.34E-07
% Males with at least a bachelor degree							-57.53	5.082	0						
% Females with at least a bachelor degree	-28.83	4.625	5.34E-10												
Median age (Both sexes)													-22.21	4.357	3.69E-07
Dentists per 10,000 population	8.526	2.741	0.00189	-13.64	5.059	0.007062				-25.11	5.394	3.40E-06			
% Uninsured (All ages)							39.83	6.642	2.32E-09						
% People below poverty line										32.28	5.499	4.95E-09			
% People unemployed	-13.48	3.744	0.000324	-14.58	3.441	2.34E-05	-13.65	3.479	8.93E-05						
Median household income				-47.65	6.822	3.63E-12									
% Occupied housing units of total housing				14.83	4.927	0.002634	44.68	6.583	1.42E-11						
% Owner occupied housing units lacking plumbing				-15.49	4.651	0.000883							-71.16	18.65	0.000139
% Owner-Renter occupied housing with lacking plumbing				10.12	4.551	0.02633									
% Two or more races													-14.32	5.343	0.007386
% Hispanic or Latino	-18.36	2.409	3.59E-14	-32.48	4.157	8.11E-15	-48.55	4.605	0	-20.81	7.194	0.003854			
% Adults reporting no exercise	10.67	3.972	0.007298	11.19	3.974	0.004909	12.62	3.639	0.00053	16.35	3.974	4.00E-05			
% Adults reporting high blood pressure										9.657	3.519	0.006112			
% Smokers	13.24	4.007	0.000964	12.15	3.808	0.001438							12.62	4.412	0.004261
% Adults reporting diabetes													14.28	5.834	0.01442
Murder per 100,000 population	5.924	2.105	0.004921										18.67	3.768	7.78E-07
Assault per 100,000 population	11.07	3.884	0.0044												
Total suicide death per 100,000 population	12.48	1.863	2.57E-11	13.13	3.516	0.000192	13.7	4.074	0.00078	21.39	5.681	0.00017			
People employed in mining, construction, manufacturing, etc. per 10,000 population	-8.35	2.66	0.001717				-50.92	9.953	3.36E-07				-70.76	27.06	0.00899
People employed in agriculture, fishing, hunting, etc. per 10,000 population				12.61	4.423	0.004408									
