# Supplementary material for: Environmental Predictors of US County Mortality Patterns on a National Basis
Source: PLoS One. 2015 Dec 2;10(12):e0137832. doi: 10.1371/journal.pone.0137832 (PMC4668104; doi:10.1371/journal.pone.0137832)
Supplement: S12 Table — (PDF) [file pone.0137832.s022.pdf]

S12 Table. Regression Parameters Derived from Stepwise Regression Analysis of Variables for Combination of Cardiovascular Diseases, Cancers and COPD for Five Population Density Groups.

Variable	Lowest Density Quintile			Quintile 2			Quintile 3			Quintile 4			Highest Density Quintile		
	Regression coefficient	Standard deviation	P value	Regression coefficient	Standard deviation	P value	Regression coefficient	Standard deviation	P value	Regression coefficient	Standard deviation	P value	Regression coefficient	Standard deviation	P value
Intercept Term							476.3	13.8	0						
Ozone							7.738	1.195	1.14E-10						
Population density	3666	1199	0.00227												
% Single parent households							30.3	5.392	2.12E-08	14.6	3.481	2.83E-05	12.72	3.261	9.83E-05
% Married-Couple families	-15.27	2.339	7.99E-11												
% 16-64 years (Both sexes) with physical disability	5.587	2.028	0.00591	10.21	2.435	2.83E-05	10.08	2.875	0.000464	9.126	3.098	0.003251	24.03	4.132	6.81E-09
% ≥65 years (Both sexes) with mental disability	-5.191	1.489	0.0005							-11.2	3.263	0.000607			
Civic and social organizations per 10,000 population				-8.565	2.983	0.004129									
Physical fitness facilities per 10,000 population							-7.926	2.954	0.007334						
Aggregate for all of social capital variables per 10,000 population				10.13	3.42	0.003096									
Response rate from the Census				-17.99	3.588	5.67E-07									
% Votes cast for President	-15.31	2.36	1.03E-10	-13.92	3.01	3.93E-06	-10.28	2.995	0.000608	-15.63	2.473	3.08E-10	-10.1	3.072	0.001027
% Males with at least a bachelor degree				-24.17	3.786	2.05E-10	-30.37	4.349	3.67E-12				-15.09	2.252	2.52E-11
% Females with at least a bachelor degree	-19.67	3.154	5.24E-10							-19.76	3.19	6.74E-10			
Median age (Both sexes)													-9.903	2.826	0.0004661
Dentists per 10,000 population	6.585	1.883	0.00048												
% Uninsured (All ages)							22.66	4.415	3.07E-07						
% People below poverty line				20.29	3.144	1.31E-10				17.49	3.851	5.83E-06			
% People unemployed				-5.938	2.319	0.01051	-9.947	2.505	7.39E-05						
% Occupied housing units of total housing				13.59	3.218	2.50E-05									
% Owner occupied housing units lacking plumbing	-7.241	1.498	1.42E-06	-10.94	2.126	2.86E-07							-36.57	13.79	0.008043
% Owner-Renter occupied housing with lacking plumbing							-11.94	2.903	4.00E-05						
% Black or African American							-10.05	4.233	0.01771						
% Native Hawaiian and Pacific Islander	-7.37	3.083	0.0169												
% Two or more races										7.463	3.063	0.0149			
% Hispanic or Latino	-12.32	1.712	8.19E-13	-22.32	2.602	0	-34.73	3.599	0	-29.93	4.177	1.02E-12	-21.86	3.049	9.82E-13
% Adults reporting no exercise	15.82	2.81	1.99E-08	10.65	2.717	9.06E-05	11.17	2.474	6.60E-06	10.31	2.863	0.000322	11.2	3.071	0.0002711
% Adults who are obese	10.35	2.279	5.88E-06												
% Smokers	9.496	2.497	0.00015	9.811	2.684	0.000263									
Murder per 100,000 population	4.728	1.5	0.00164												
Assault per 100,000 population	10.11	2.758	0.00025												
Total suicide death per 100,000 population	5.879	1.311	7.60E-06												
People employed in mining, construction, manufacturing, etc. per 10,000 population				-14.76	5.01	0.003241	-27.31	6.681	4.48E-05						
People employed in agriculture, fishing, hunting, etc. per 10,000 population	8.945	3.019	0.00308												
