# Supplementary material for: Environmental Predictors of US County Mortality Patterns on a National Basis
Source: PLoS One. 2015 Dec 2;10(12):e0137832. doi: 10.1371/journal.pone.0137832 (PMC4668104; doi:10.1371/journal.pone.0137832)
Supplement: S13 Table — (PDF) [file pone.0137832.s023.pdf]

# S13 Table. Regression Parameters Derived from Stepwise Regression Analysis of Variables for Cancers for Five Population Density Groups.

| Variable | Lowest Density Quintile | | | Quintile 2 | | | Quintile 3 | | | Quintile 4 | | | Highest Density Quintile | | |
|---|---|---|---|---|---|---|---|---|---|---|---|---|---|---|---|
| | Regression coefficient | Standard deviation | P value | Regression coefficient | Standard deviation | P value | Regression coefficient | Standard deviation | P value | Regression coefficient | Standard deviation | P value | Regression coefficient | Standard deviation | P value |
| Intercept Term | | | | | | | 113.8 | 3.459 | 0 | | | | | | |
| Ozone | | | | | | | 0.8832 | 0.3956 | 0.02568 | | | | | | |
| % Rural population | -2.748 | 1.015 | 0.006833 | | | | | | | | | | | | |
| % Foreign-born population | | | | | | | | | | | | | -3.295 | 0.5548 | 3.26E-09 |
| % Single parent households | | | | | | | 11.42 | 1.667 | 9.39E-12 | 6.547 | 1.254 | 1.94E-07 | | | |
| % Married-Couple families | -4.354 | 0.8182 | 1.12E-07 | | | | | | | | | | | | |
| % 16-64 years (Both sexes) with physical disability | 1.612 | 0.6684 | 0.01593 | 2.116 | 0.7857 | 0.007122 | 3.256 | 0.7928 | 4.13E-05 | 5.311 | 1.004 | 1.33E-07 | 6.67 | 1.315 | 4.25E-07 |
| % ≥65 years (Both sexes) with physical disability | 2.745 | 0.6437 | 2.08E-05 | | | | | | | | | | | | |
| Physical fitness facilities per 10,000 population | | | | | | | | | | | | | 3.654 | 0.9677 | 0.0001628 |
| Professional organizations per 10,000 population | 1.224 | 0.4999 | 0.01443 | | | | | | | | | | | | |
| Memberships in organizations not classified elsewhere per 10,000 population | -1.297 | 0.3885 | 0.000853 | | | | | | | | | | | | |
| Non-profit organizations per 10,000 population | -2.237 | 0.6976 | 0.00136 | | | | | | | | | | | | |
| % Votes cast for President | -2.231 | 0.8439 | 0.008261 | | | | | | | | | | | | |
| % Males with at least a bachelor degree | -6.787 | 1.5 | 6.34E-06 | | | | -6.383 | 1.044 | 1.14E-09 | | | | | | |
| % Females with at least a bachelor degree | | | | -5.913 | 0.9421 | 4.07E-10 | | | | | | | | | |
| Dentists per 10,000 population | | | | | | | | | | -3.813 | 1.121 | 0.000681 | | | |
| % People below poverty line | | | | 6.32 | 0.7566 | 1.11E-16 | | | | 3.697 | 1.208 | 0.002233 | | | |
| % People unemployed | | | | | | | -2.754 | 0.7998 | 0.000584 | -2.198 | 0.8833 | 0.01291 | | | |
| Per capita income (Total population) | 7.507 | 1.275 | 4.45E-09 | | | | | | | | | | | | |
| % Occupied housing units of total housing | 2.514 | 0.6229 | 5.59E-05 | | | | | | | | | | | | |
| % Owner occupied housing units lacking plumbing | -1.319 | 0.5131 | 0.0102 | -2.996 | 0.6896 | 1.45E-05 | | | | | | | | | |
| % Black or African American | | | | | | | -3.531 | 1.32 | 0.007528 | | | | 5.327 | 0.7886 | 1.77E-11 |
| % Hispanic or Latino | -6.073 | 0.5901 | 0 | -6.705 | 0.7972 | 1.11E-16 | -8.654 | 0.8942 | 0 | -6.451 | 1.109 | 6.77E-09 | | | |
| Maximum temperature in August | | | | | | | | | | | | | 1.862 | 0.7092 | 0.008711 |
| % Adults reporting no exercise | 2.443 | 0.9521 | 0.01034 | | | | 3.342 | 0.7842 | 2.11E-05 | | | | | | |
| % Adults reporting an average of fruit and vegetables consumption of less than 5 servings per day | | | | | | | | | | -2.686 | 0.4977 | 7.40E-08 | -2.44 | 0.6135 | 7.16E-05 |
| % Adults who are obese | | | | | | | | | | 3.043 | 0.8789 | 0.000545 | | | |
| % Smokers | 4.706 | 0.816 | 9.00E-09 | 3.981 | 0.769 | 2.43E-07 | | | | 2.703 | 0.8739 | 0.001998 | 4.176 | 1.069 | 9.53E-05 |
| % Adults reporting diabetes | 2.355 | 0.667 | 0.000422 | | | | | | | | | | | | |
| Murder per 100,000 population | 1.293 | 0.4945 | 0.008964 | | | | | | | | | | | | |
| Assault per 100,000 population | | | | 2.965 | 0.9232 | 0.001336 | | | | | | | | | |
