# Supplementary material for: Environmental Predictors of US County Mortality Patterns on a National Basis
Source: PLoS One. 2015 Dec 2;10(12):e0137832. doi: 10.1371/journal.pone.0137832 (PMC4668104; doi:10.1371/journal.pone.0137832)
Supplement: S14 Table — (PDF) [file pone.0137832.s024.pdf]

S14 Table. Regression Parameters Derived from Stepwise Regression Analysis of Variables for COPD for Five Population Density Groups.

Variable	Lowest Density Quintile			Quintile 2			Quintile 3			Quintile 4			Highest Density Quintile		
	Regression coefficient	Standard deviation	P value	Regression coefficient	Standard deviation	P value	Regression coefficient	Standard deviation	P value	Regression coefficient	Standard deviation	P value	Regression coefficient	Standard deviation	P value
Intercept Term							37.81	2.165	0						
Population density	-1228	279.5	1.17E-05												
% Rural population	-2.922	0.6881	2.25E-05												
% Foreign-born population	1.422	0.5985	0.01761										-2.097	0.357	4.78E-09
% Single parent households	3.364	0.7669	1.20E-05	7.447	1.114	2.83E-11	7.031	1.088	1.22E-10	4.997	0.9763	3.32E-07			
% 16-64 years (Both sexes) with physical disability	2.511	0.4881	2.87E-07	2.911	0.5194	2.32E-08	3.213	0.5276	1.30E-09	4.73	0.5362	0	5.497	0.8892	7.38E-10
% ≥65 years (Both sexes) with physical disability	1.99	0.4846	4.14E-05												
% ≥65 years (Both sexes) with mental disability	-0.9293	0.3644	0.01082												
Public golf courses per 10,000 population	0.9864	0.2863	0.000579												
Aggregate for all of social capital variables per 10,000 population	-1.771	0.4072	1.43E-05				-1.824	0.7414	0.01397						
% Votes cast for President				-1.659	0.6186	0.007374	-3.097	0.5526	2.33E-08	-2.749	0.4504	1.20E-09			
Median age (Both sexes)													-2.808	0.5274	1.10E-07
Dentists per 10,000 population	1.42	0.4808	0.00318												
% People below poverty line	-5.336	0.8035	3.81E-11												
Per capita income (Total population)				-3.281	0.7812	2.76E-05									
% Occupied housing units of total housing	1.475	0.5136	0.004126												
% Owner-Renter Occupied housing with lacking plumbing	-1.566	0.4966	0.001639												
% Black or African American				-9.377	0.9904	0	-8.559	0.9084	0	-6.573	0.8778	9.57E-14	-2.716	0.5506	8.64E-07
% American Indian and Alaska Native				-3.704	0.6543	1.67E-08									
% Some other race				7.058	1.53	4.15E-06									
% Hispanic or Latino				-9.288	1.624	1.20E-08	-4.628	0.6386	5.60E-13						
Average annual precipitation	-2.015	0.501	5.93E-05												
% Adults reporting no exercise				1.265	0.5152	0.01416	1.749	0.5106	0.000622						
% Adults reporting an average of fruit and vegetables consumption of less than 5 servings per day	-1.881	0.5502	0.000641												
% Adults who are obese	2.032	0.5162	8.47E-05												
% Smokers	4.373	0.6394	9.89E-12										1.789	0.6855	0.00912
Assault per 100,000 population	3.084	0.6458	1.89E-06												
Total suicide death per 100,000 population				1.887	0.5552	0.000689									
People employed in mining, construction, manufacturing, etc. per 10,000 population	1.606	0.5206	0.002055	-3.659	1.019	0.000338				-5.353	1.895	0.004776			
