# Supplementary material for: Environmental Predictors of US County Mortality Patterns on a National Basis
Source: PLoS One. 2015 Dec 2;10(12):e0137832. doi: 10.1371/journal.pone.0137832 (PMC4668104; doi:10.1371/journal.pone.0137832)
Supplement: S11 Table — (PDF) [file pone.0137832.s021.pdf]

S11 Table. Regression Parameters Derived from Stepwise Regression Analysis of Variables for Cardiovascular Diseases for Five Population Density Groups.

Variable	Lowest Density Quintile			Quintile 2			Quintile 3			Quintile 4			Highest Density Quintile		
	Regression coefficient	Standard deviation	P value	Regression coefficient	Standard deviation	P value	Regression coefficient	Standard deviation	P value	Regression coefficient	Standard deviation	P value	Regression coefficient	Standard deviation	P value
Intercept Term							318.6	11.04	0						
PM_{10}							1.726	0.7639	0.02394						
Ozone							7.552	0.9862	2.67E-14						
Population density	2767	1030	0.007271												
% Foreign-born population				-8.764	3.469	0.01159									
% Single parent households	16.6	3.503	2.26E-06	10.74	2.778	0.000114	15.74	2.72	8.18E-09	9.347	3.116	0.002732	8.379	3.068	0.006357
% Married-Couple families	-6.606	2.262	0.003526												
% 16-64 years (Both sexes) with employment disability				-7.038	1.939	0.00029									
% ≥65 years (Both sexes) with mental disability	-3.878	1.225	0.001559												
Religious organizations per 10,000 population	4.449	1.329	0.000824				7.103	2.437	0.003594						
Professional organizations per 10,000 population	-4.593	1.226	0.000183												
Non-profit organizations per 10,000 population							-10.96	2.974	0.000232						
Response rate from the Census				-10.5	1.869	2.16E-08									
% Votes cast for President	-11.26	1.953	9.12E-09	-12.15	2.294	1.27E-07				-9.241	2.301	6.07E-05	-7.698	2.21	0.000504
% Males with at least a bachelor degree	-18.59	2.921	2.33E-10	-13.08	3.137	3.16E-05	-15.84	3.986	7.27E-05				-14.52	1.754	2.22E-16
% Females with at least a bachelor degree										-14.86	2.531	4.98E-09			
Median age (Both sexes)										-6.612	2.132	0.001948			
Dentists per 10,000 population	6.8	1.586	1.88E-05												
% Uninsured (All ages)							12.62	4.037	0.001791	15.53	3.83	5.16E-05	14.04	4.626	0.002434
% People below poverty line				15.71	3.41	4.29E-06									
% People unemployed				-7.063	1.973	0.00035	-4.814	1.956	0.01391						
Median household income							-16.98	3.368	4.92E-07						
% Occupied housing units of total housing	-8.889	1.766	5.15E-07												
% Owner occupied housing units lacking plumbing				-8.123	1.728	2.73E-06	-16.14	3.602	7.81E-06						
% Owner-Renter occupied housing lacking plumbing	-5.683	1.699	0.000832												
% American Indian and Alaska Native	-4.959	1.975	0.0121												
% Two or more races				5.794	1.857	0.001824	4.428	2.01	0.02772						
% Hispanic or Latino	-7.496	1.803	3.34E-05	-11.89	3.154	0.000168	-17.68	2.477	1.25E-12	-24.47	3.147	1.08E-14	-21.42	3.034	2.12E-12
% Adults reporting no exercise	13.81	2.306	2.41E-09	9.639	2.173	9.58E-06	6.876	1.986	0.000543	8.057	2.201	0.000257	8.173	2.618	0.001821
% Adults reporting high blood pressure				5.142	1.88	0.006277							-5.812	2.203	0.008381
% Adults who are obese	5.322	1.943	0.006215												
Murder per 100,000 population	2.923	1.208	0.01556												
Total suicide death per 100,000 population	5.224	1.071	1.13E-06												
People employed in mining, construction, manufacturing, etc. per 10,000 population							-20.83	5.461	0.00014						
People employed in agriculture, fishing, hunting, etc. per 10,000 population	9.888	2.604	0.00015	14.1	2.49	1.67E-08									
