# Supplementary figures and images for: Correction: Environmental Predictors of US County Mortality Patterns on a National Basis
Source: PLoS One. 2016 Jan 4;11(1):e0146506. doi: 10.1371/journal.pone.0146506 (PMC4699811; doi:10.1371/journal.pone.0146506)

S1 Fig.

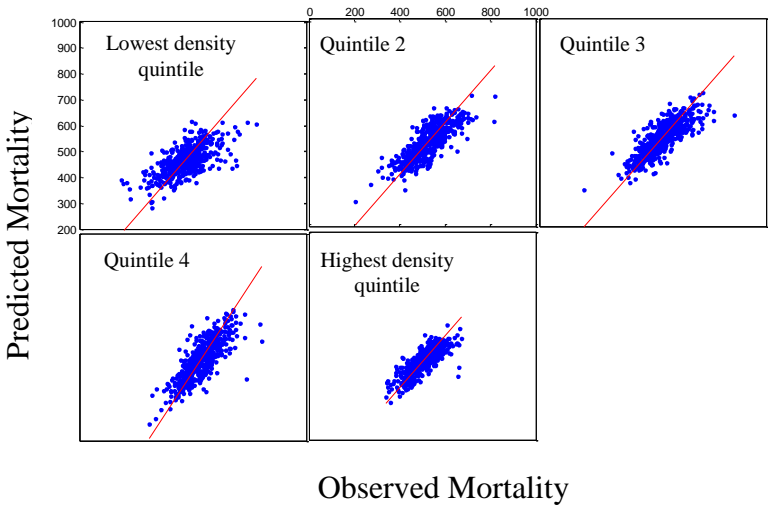

Supplement: S1 Fig — Observed versus estimated mortality in 2,591 counties in the prediction set (Set 1) using stepwise regression for five population density groups (R-squared = 0.6494). (PDF) [file pone.0146506.s001.pdf]

S2 Fig.

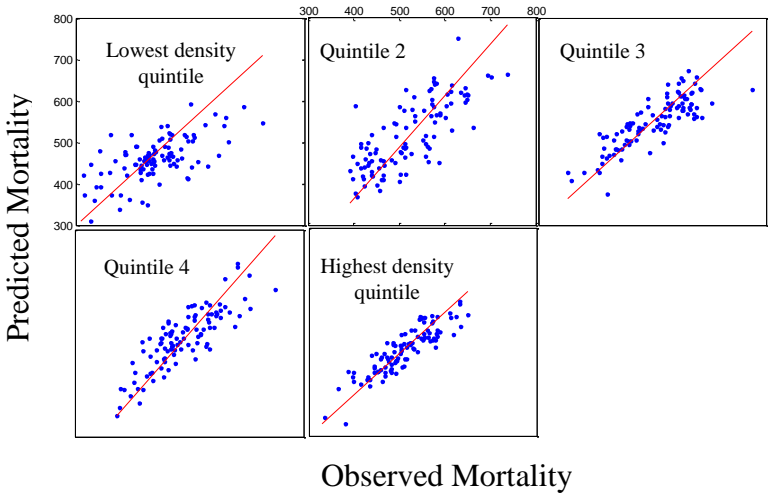

Supplement: S2 Fig — Observed versus estimated mortality in 519 counties in the validation set (Set 2) using stepwise regression for five population density groups. (PDF) [file pone.0146506.s002.pdf]

S3 Fig.

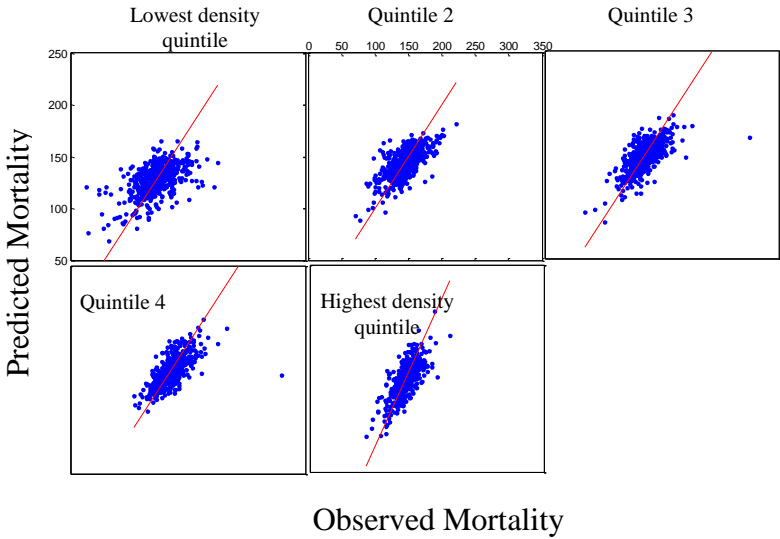

Supplement: S3 Fig — Observed versus estimated mortality in 2,591 counties in the prediction set (Set 1) using stepwise regression for five population density groups (R-squared = 0.4928). (PDF) [file pone.0146506.s003.pdf]

S4 Fig.

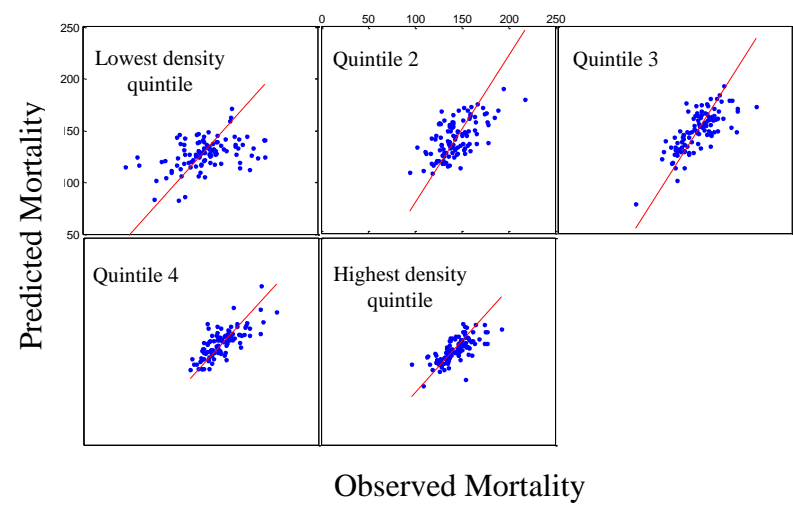

Supplement: S4 Fig — Observed versus estimated mortality in 519 counties in the validation set (Set 2) using stepwise regression for five population density groups. (PDF) [file pone.0146506.s004.pdf]

S5 Fig.

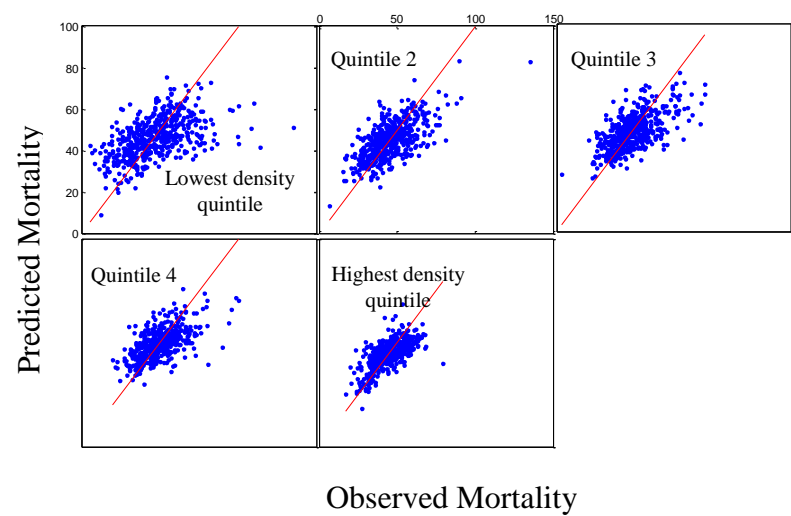

Supplement: S5 Fig — Observed versus estimated mortality in 2,591 counties in the prediction set (Set 1) using stepwise regression for five population density groups (R-squared = 0.3732). (PDF) [file pone.0146506.s005.pdf]

S6 Fig.

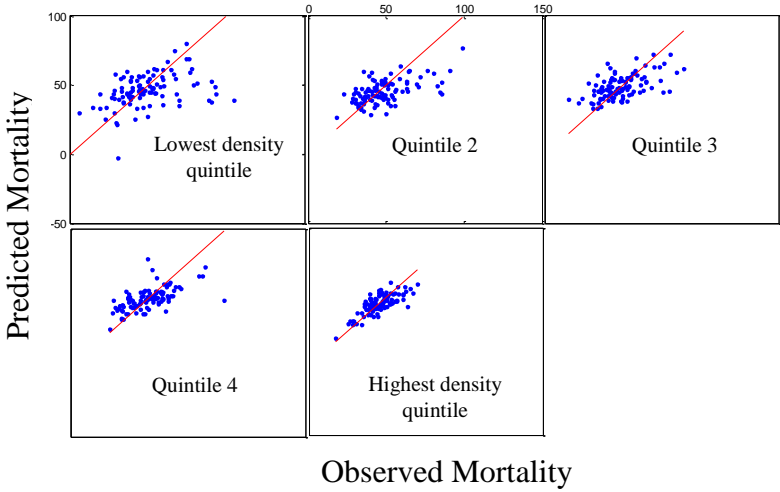

Supplement: S6 Fig — Observed versus estimated mortality in 519 counties in the validation set (Set 2) using stepwise regression for five population density groups. (PDF) [file pone.0146506.s006.pdf]
